# Surveillance for Cancer Incidence and Mortality — United States, 2013

**DOI:** 10.15585/mmwr.ss6604a1

**Published:** 2017-01-27

**Authors:** Simple D. Singh, S. Jane Henley, A. Blythe Ryerson

**Affiliations:** 1 Division of Cancer Prevention and Control, National Center for Chronic Disease Prevention and Health Promotion, CDC

## Preface

This report provides, in tabular and graphic form, official federal statistics on cancer incidence and mortality for 2013 and trends for 1999–2013 as reported by CDC and the National Cancer Institute (NCI). Data in this report come from the United States Cancer Statistics (USCS) system (*1*), which includes cancer incidence data from population-based cancer registries that participate in CDC’s National Program of Cancer Registries (NPCR) and NCI’s Surveillance, Epidemiology, and End Results (SEER) program reported as of November 2015 and cancer mortality data from death certificate information reported to state vital statistics offices as of June 2015 and compiled into a national file for the entire United States by CDC’s National Center for Health Statistics (NCHS) National Vital Statistics System (NVSS).

This report presents information on new cancer cases and deaths for 2013. The number and rate of cancer cases and deaths are stratified by the primary cancer sites as reported for 2013; information is provided by demographic characteristic (e.g., sex, age, race, and ethnicity) and primary cancer site (68 selected sites among men and 72 selected sites among women). Age-adjusted cancer incidence and death rates are shown by primary site and year for the period 1999–2013. Age-adjusted cancer incidence and death rates for the most common sites are shown by race, sex, and ethnicity for 2013, the most recent diagnosis year for which incidence data are available. Maps of the United States display age-adjusted cancer incidence and death rates, presented by quartiles, for 2013. Time trends in age-adjusted cancer incidence and death rates during 1999–2013 are shown for all sites combined, colorectal, lung and bronchus, prostate, and female breast by race, sex, and ethnicity.

## Background

Cancer comprises a diverse mix of diseases occurring in every part of the body and is a leading cause of death in the United States (*2*). More than half of cancer cases could be prevented (*3*). Surveillance of cancer incidence and mortality can help public health officials target areas for control efforts (*4*) and track progress toward meeting the national health objectives set forth in *Healthy People 2020* (*5*). As of 2016, *Healthy People 2020* objectives included reducing cancer deaths per 100,000 persons to 161.4 for all cancers, 45.5 for lung cancer, 20.7 for female breast cancer, 2.2 for cervical cancer, 14.5 for colorectal cancer, 2.3 for oropharyngeal cancer, 21.8 for prostate cancer, 2.4 for melanoma and reducing cancer incidence per 100,000 persons to 39.9 for colorectal cancer, 7.2 for cervical cancer, and 42.1 for late-stage female breast cancer (*5*).

Cancer is a reportable disease in every state and thus all hospitals, physicians’ offices, pathology laboratories, and other medical facilities are required to submit data on all reportable cancer diagnoses to a central cancer registry at the state or territorial level. A cancer registry is a database that contains individual records of all reportable cancer cases in a defined population and includes patient demographics, tumor characteristics (e.g., cancer site and pathology), and information about the notifying health provider or facility. Cancer control planners and others can identify variations in cancer rates by population subgroups and monitor trends over time to guide the planning and evaluation of cancer prevention and control programs and allocation of health resources.

## Data Sources

Data about cancer incidence and mortality come from the official federal statistics on cancer, the USCS dataset (*1*). The USCS dataset includes cancer incidence data from NPCR registries in 45 states and the District of Columbia (DC) (cancer incidence data from Puerto Rico and the U.S. Pacific Island Jurisdictions were not available for this analysis) and from SEER program registries in the remaining five states (Connecticut, Hawaii, Iowa, New Mexico, and Utah) and cancer mortality data from NVSS. Incidence data included in USCS have met publication criteria.

### Incidence Data

The primary source of data on cancer incidence is medical records. Staff at medical facilities such as hospitals, doctors’ offices, and pathology laboratories abstract data from patients’ medical records, enter it into the facility’s own cancer registry if it has one, and then send the data to the regional or state registry. The data then are sent to the central cancer registry in that state, district or territory. Every year the central cancer registries electronically submit incidence, demographic, and clinical data to NPCR or SEER. Both NPCR and SEER registries collect data using uniform data items and codes as documented by the North American Association of Central Cancer Registries (NAACCR). This uniformity ensures that data items collected by the two federal programs are comparable (*6*,*7*). Information on primary site and histology is coded according to the International Classification of Diseases for Oncology, Third Edition (ICD-O-3) and categorized according to the revised SEER recodes dated January 27, 2003, which define standard groupings of primary cancer sites (https://seer.cancer.gov/siterecode) (*8*). Beginning with 2010 diagnoses, cases were first classified by anatomic site by using ICD-O-3; cases with hematopoetic histologies were further classified by using the 2008 World Health Organization (WHO) Classification of Tumours of Haematopoietic and Lymphoid Tissues (*9*). Data from the NPCR registries provided in this report were reported to CDC as of November 30, 2015. Data from SEER registries were reported to NCI as of November 1, 2015.

NPCR and SEER cancer registries consider as reportable all incident cases with a behavior code of 2 (in situ, noninvasive) or 3 (malignant, primary site only) in ICD-O-3. Exceptions include in situ cancer of the cervix and all basal and squamous cell carcinomas of the skin, except for those on the skin of the genital organs (*8*). Beginning with 2001 diagnoses, several cancers that are coded as malignant in ICD-O-3 were not coded as malignant in ICD-O-2 (*6*). Additional information is provided in the USCS technical notes (https://www.cdc.gov/cancer/npcr/uscs/pdf/uscs-2013-technical-notes.pdf#nameddest=RegistriesPubCriteria#nameddest=IncidenceDataSources).

### Mortality Data

Cancer mortality statistics are based on information from all death certificates filed in the 50 states and DC and processed by NVSS at NCHS (*10*). The cancer mortality data were compiled in accordance with WHO regulations, which specify that member nations classify and code causes of death in accordance with the current revision of the International Classification of Diseases (ICD) (*11*). For consistency with the data on cancer incidence, the cancer sites in mortality data were grouped according to the revised SEER recodes dated January 27, 2003 (https://seer.cancer.gov/codrecode). Data for a specific calendar year are based on records of deaths that occurred during that calendar year and received by a particular date. Data in this report include, mortality data for 2013 based on records of deaths that occurred during 2013 and received by NCHS as of June 30, 2015. Data in this report come from USCS, which includes cancer deaths during 1998–2013; cancer mortality data for 2014 are available at https://www.cdc.gov/nchs/nvss/deaths.htm. Additional information about mortality data is provided in the USCS technical notes (https://www.cdc.gov/cancer/npcr/uscs/pdf/uscs-2013-technical-notes.pdf#nameddest=RegistriesPubCriteria#nameddest=MortalityDataSources).

### Population Estimates

Population denominators are annual race-specific, ethnicity-specific, and sex-specific county population estimates modified by NCI in collaboration with CDC’s NCHS from the U.S. Census intercensal (for July 1, 1999–2009) and Vintage 2014 (for July 1, 2010–2013) annual times series (*12*). Modifications incorporated bridged single-race estimates that are derived from the original multiple race categories in the 2000 and 2010 censuses. For most states, population estimates as of July 1 of each year were used to calculate annual incidence rates because these estimates are presumed to reflect the average population of a defined geographic area for a calendar year. However, some county population estimates were adjusted to account for populations displaced along the Gulf Coast of Louisiana, Alabama, Mississippi, and Texas in the fall of 2005 by hurricanes Katrina and Rita. The national total population estimates were not affected by these adjustments. Other specific modifications included using additional local information to accurately estimate the native Hawaiian population and deriving population estimates for newly created counties in Colorado and Alaska. The modified county-level population estimates, summed to the state and national level, were used as denominators in rate calculations. Additional details about population data are available at https://seer.cancer.gov/popdata/index.html.

## Publication Criteria

Cancer incidence data are derived from state cancer registries that have high-quality cancer incidence data for individual (e.g., 2013) and combined (e.g., 1999–2013) years as demonstrated by meeting all of the following criteria on data quality for all cancer sites combined:

- case ascertainment is ≥90% (margin of error +5%) complete,
- ≤5% of cases are ascertained solely on the basis of a death certificate,
- ≤3% of cases are missing information on sex,
- ≤3% of cases are missing information on age,
- ≤5% of cases are missing information on race, and
- ≥97% of the registry’s records passed a set computerized edits that test the validity and logic of data components.

In this report, cancer incidence data for 2013 include data from DC and all states except Nevada, covering 99% of the US population, and data for 1999–2013 include data from all registries except Arkansas, DC, Mississippi, Nevada, South Dakota, Tennessee, and Virginia, covering 92% of the U.S. population. Additional information about USCS is available at available at https://www.cdc.gov/uscs/.

## Interpreting Data

### Incidence Data

Each year, state cancer registries submit cancer cases for a new diagnosis year and an updated version of the previous year’s cancer cases to CDC or NCI. Therefore, each year, when USCS data are published, updates to the previous year’s data are published, using the most recent data submission and the most recent population data. Users of cancer incidence data published by federal agencies need to be mindful of the date of data submission for data used in their analyses.

### Mortality Data

Cancer mortality statistics in USCS are influenced by the accuracy of information on the death certificate. Unlike incidence data, mortality data for a calendar year are considered complete when submitted and so are not updated after the aggregate data file is released. Mortality data for the entire United States refer only to deaths that occurred within the United States; data for geographic areas are provided by the decedent’s place of residence.

### Race and Ethnicity Data

Differences in rates among racial and ethnic populations should be interpreted with caution. For cancer incidence, race and ethnicity data are abstracted from medical records and grouped into categories (*7*). A study using SEER incidence data suggests that the quality of data on race/ethnicity in cancer registries is considered excellent for whites, blacks, and Asians/Pacific Islanders, good for Hispanics, and poor for American Indians/Alaska Natives (*13*). When cancer mortality is reported, race and ethnic origin are recorded separately on the death certificate by the funeral director as provided by an informant or, in the absence of an informant, on the basis of observation (*14*). Previous studies involving cancer mortality data demonstrate that death rates for whites and blacks generally are estimated accurately whereas death rates for Asians/Pacific Islanders, American Indians/Alaska Natives, and Hispanics are underestimated (*2*,*15*). Recent study involving evaluation of validity of race showed that classification of race and ethnicity on death certificates improved overtime for Asian/Pacific Islanders and Hispanics (now almost as good as whites and blacks), however, remained poor for American Indians/Alaska Natives (*16*). For this reason, incidence and mortality data provided in this report might be underestimated for American Indians/Alaska Natives groups, possibly because of misclassification of race.

Three NPCR registries (Delaware, Kansas, and Kentucky) opted not to present state-specific Asian/Pacific Islander counts and rates. Six NPCR registries (Delaware, Kentucky, Massachusetts, Pennsylvania, North Dakota, and Virginia) opted not to present state-specific Hispanic (classified by the NAACCR Hispanic Identification [NHIA] Algorithm) counts and rates (*17*). Cancer registries regularly link their database to the Indian Health Service patient registration dataset to reduce misclassification of race for American Indian/Alaska Native patients; in this report, 34 NPCR registries and all SEER registries linked cases diagnosed in 1999–2013. Six NPCR registries (Delaware, Illinois, Kansas, Kentucky, New Jersey, and New York) opted not to present state-specific American Indian/Alaska Native counts and rates. However, the aggregate national rates presented in this report include data for these registries, except that incidence rates by ethnicity exclude Virginia because ethnicity information was missing on 85% of cases from Virginia.

### Population Coverage

The population coverage for incidence data varies by diagnosis year. Population coverage might be affected by the suppression of state incidence data if a state did not meet the publication criteria or did not submit data for that diagnosis year. In addition, state incidence data were suppressed in this report if <16 cases were reported or if the state requested that the data be suppressed. Additional information is provided by the USCS technical notes (https://www.cdc.gov/cancer/npcr/uscs/pdf/uscs-2013-technical-notes.pdf#nameddest=RegistriesPubCriteria#nameddest=CensusRegionPubCriteria). Mortality data from malignant neoplasms (i.e., cancers) as recorded in NVSS from the 50 states and DC are available in USCS, and thus 100% of the U.S. population is covered each year. However, state death data were suppressed in this report if <16 deaths were reported.

### Suppression of Rates and Counts

When the numbers of cases or deaths used to compute rates are small, those rates tend to have poor reliability. Therefore, incidence and death rates and counts of <16 are not shown in tables and figures. The use of a threshold value for suppressing cells helps protect the confidentiality of patients by reducing or eliminating the risk for disclosure of their identity and helps avoid overreliance on unstable data. Additional information is provided in the USCS technical notes (https://www.cdc.gov/cancer/npcr/uscs/pdf/uscs-2013-technical-notes.pdf#nameddest=RegistriesPubCriteria#nameddest=Suppression).

## Highlights

### Incidence and Death Rates

In 2013, more than 1.5 million invasive cancers were diagnosed in the United States, an annual incidence rate of 439 cases per 100,000 persons (Table 1). In the same year, approximately 584,872 persons died of cancer nationally, an annual death rate of 163 deaths per 100,000 persons (Table 2). Overall and for many cancer sites, males had higher incidence (Table 1) and death rates (Table 2) than did females.

**TABLE 1:** Reported number and rate* of invasive^†^ cancer cases, by primary cancer site and sex — United States, 2013^§^

Cancer site	Male		Female		Total	
	No.	Rate	No.	Rate	No.	Rate
**All sites combined**	**781,451**	**479.0**	**777,679**	**412.6**	**1,559,130**	**439.0**
**Oral cavity and pharynx**	**29,693**	**17.4**	**12,024**	**6.3**	**41,717**	**11.5**
Lip	1,399	0.9	547	0.3	**1,946**	**0.6**
Tongue	9,164	5.3	3,592	1.9	**12,756**	**3.5**
Salivary gland	2,629	1.7	1,779	1.0	**4,408**	**1.3**
Floor of mouth	1,368	0.8	621	0.3	**1,989**	**0.5**
Gum and other mouth	3,054	1.9	2,392	1.2	**5,446**	**1.5**
Nasopharynx	1,289	0.8	521	0.3	**1,810**	**0.5**
Tonsil	6,528	3.7	1,415	0.7	**7,943**	**2.1**
Oropharynx	1,459	0.8	447	0.2	**1,906**	**0.5**
Hypopharynx	1,893	1.1	450	0.2	**2,343**	**0.6**
Other oral cavity and pharynx	910	0.5	260	0.1	**1,170**	**0.3**
**Digestive system**	**155,535**	**95.4**	**124,185**	**63.8**	**279,720**	**78.2**
Esophagus	12,913	7.8	3,519	1.8	**16,432**	**4.5**
Stomach	14,429	9.0	8,719	4.5	**23,148**	**6.6**
Small intestine	4,307	2.6	3,887	2.0	**8,194**	**2.3**
Colon and rectum	71,099	44.2	65,020	33.6	**136,119**	**38.4**
Colon excluding rectum	48,175	30.3	48,748	25.0	**96,923**	**27.4**
Rectum and rectosigmoid junction	22,924	13.9	16,272	8.6	**39,196**	**11.0**
Anus, anal canal, and anorectum	2,441	1.5	4,136	2.1	**6,577**	**1.8**
Liver and intrahepatic bile duct	21,143	12.0	8,330	4.2	**29,473**	**7.9**
Gallbladder	1,280	0.8	2,654	1.3	**3,934**	**1.1**
Other biliary	3,401	2.2	2,979	1.5	**6,380**	**1.8**
Pancreas	22,787	14.1	21,590	10.9	**44,377**	**12.4**
Retroperitoneum	623	0.4	610	0.3	**1,233**	**0.4**
Peritoneum, omentum, and mesentery	170	0.1	1,804	0.9	**1,974**	**0.6**
Other digestive organs	942	0.6	937	0.5	**1,879**	**0.5**
**Respiratory system**	**123,760**	**76.9**	**104,348**	**53.4**	**228,108**	**63.6**
Nose, nasal cavity, and middle ear	1,435	0.9	895	0.5	**2,330**	**0.7**
Larynx	9,923	5.9	2,519	1.3	**12,442**	**3.4**
Lung and bronchus	111,907	69.8	100,677	51.5	**212,584**	**59.4**
Pleura	57	0	48	0	**105**	**0**
Trachea, mediastinum, and other respiratory organs	438	0.3	209	0.1	**647**	**0.2**
**Bones and joints**	**1,650**	**1.1**	**1,334**	**0.8**	**2,984**	**0.9**
**Soft tissue including heart**	**6,093**	**3.9**	**4,965**	**2.8**	**11,058**	**3.3**
**Skin excluding basal and squamous**	**45,977**	**29.0**	**31,938**	**17.6**	**77,915**	**22.4**
Melanoma of the skin	42,430	26.6	29,513	16.3	**71,943**	**20.7**
Other nonepithelial skin	3,547	2.4	2,425	1.3	**5,972**	**1.7**
**Male and female breast**	**NA**	**NA**	**NA**	**NA**	**232,924**	**66.3**
Female breast	NA	NA	230,815	123.7	**NA**	**NA**
Male breast	2,109	1.3	NA	NA	**NA**	**NA**
**Female genital system**	**NA**	**NA**	**91,872**	**48.6**	**NA**	**NA**
Cervix	NA	NA	11,955	7.2	**NA**	**NA**
Corpus and uterus, NOS	NA	NA	50,560	25.9	NA	NA
Corpus	NA	NA	48,937	25.0	NA	NA
Uterus, NOS	NA	NA	1,623	0.8	NA	NA
Ovary	NA	NA	20,927	11.2	NA	NA
Vagina	NA	NA	1,266	0.6	NA	NA
Vulva	NA	NA	4,895	2.6	NA	NA
Other female genital organs	NA	NA	2,269	1.2	NA	NA
**Male genital system**	**186,509**	**108.2**	**NA**	**NA**	**NA**	**NA**
Prostate	176,450	101.6	NA	NA	NA	NA
Testis	8,351	5.5	NA	NA	NA	NA
Penis	1,334	0.9	NA	NA	NA	NA
Other male genital organs	374	0.2	NA	NA	NA	NA
**Urinary system**	**91,832**	**58.0**	**39,163**	**20.3**	**130,995**	**36.9**
Urinary bladder	53,921	34.9	17,030	8.6	70,951	20.0
Kidney and renal pelvis	35,914	21.7	21,034	11.2	56,948	16.0
Ureter	1,185	0.8	722	0.4	1,907	0.5
Other urinary organs	812	0.5	377	0.2	1,189	0.3
**Eye and orbit**	**1,540**	**1.0**	**1,271**	**0.7**	**2,811**	**0.8**
**Brain and other nervous system**	**12,092**	**7.6**	**9,756**	**5.5**	**21,848**	**6.5**
Brain	11,453	7.2	9,036	5.1	20,489	6.0
Cranial nerves other nervous system	639	0.4	720	0.4	1,359	0.4
**Endocrine system**	**13,052**	**8.1**	**37,033**	**22.2**	**50,085**	**15.3**
Thyroid	11,816	7.3	35,877	21.6	47,693	14.6
Other endocrine including thymus	1,236	0.8	1,156	0.7	2,392	0.7
**Lymphomas**	**40,056**	**25.3**	**32,899**	**17.6**	**72,955**	**21.1**
Hodgkin lymphoma	4,574	2.9	3,659	2.3	8,233	2.6
Non-Hodgkin lymphoma	35,482	22.4	29,240	15.4	64,722	18.5
**Myeloma**	**12,556**	**7.8**	**10,006**	**5.1**	**22,562**	**6.3**
**Leukemias**	**26,337**	**16.9**	**19,023**	**10.3**	**45,360**	**13.2**
Acute lymphocytic leukemia	2,599	1.7	2,052	1.3	4,651	1.5
Chronic lymphocytic leukemia	9,223	5.8	5,869	3.0	15,092	4.2
Acute myeloid leukemia	7,921	5.1	6,419	3.5	14,340	4.2
Chronic myeloid leukemia	3,499	2.3	2,495	1.4	5,994	1.8
Other leukemias	3,095	2.0	2,188	1.1	5,283	1.5
**Mesothelioma**	**2,441**	**1.6**	**766**	**0.4**	**3,207**	**0.9**
**Kaposi Sarcoma**	**985**	**0.7**	**120**	**0.1**	**1,105**	**0.4**
**Miscellaneous**	**29,234**	**19.0**	**26,161**	**13.3**	**55,395**	**15.7**

**Abbreviations:** NA = not applicable; NOS = not otherwise specified.

*Rates are the number of cases per 100,000 persons and are age-adjusted to the 2000 U.S. standard population (19 age groups – Census P25–1130). For more information, see USCS technical notes (https://www.cdc.gov/cancer/npcr/uscs/pdf/uscs-2013-technical-notes.pdf).

^†^Invasive cancer excludes basal and squamous cell carcinomas of the skin except when these occur on the skin of the genital organs, and in situ cancers except urinary bladder. Urinary bladder cancer includes invasive and in situ.

^§^Data are compiled from cancer registries that meet the data quality criteria for all invasive cancer sites combined (all registries except Nevada, covering approximately 99% of the U.S. population).

**TABLE 2:** Reported number and rate* of cancer deaths, by primary cancer site and sex — United States, 2013^†^

Cancer site	Male		Female		Total	
	No.	Rate	No.	Rate	No.	Rate
**All sites combined**	**307,553**	**196.2**	**277,319**	**139.1**	**584,872**	**163.0**
Oral cavity and pharynx	6,227	3.7	2,623	1.3	**8,850**	**2.4**
Lip	42	0	17	0	**59**	**0**
Tongue	1,507	0.9	701	0.4	**2,208**	**0.6**
Salivary gland	599	0.4	287	0.1	**886**	**0.2**
Floor of mouth	51	0	33	0	**84**	**0**
Gum and other mouth	706	0.4	542	0.3	**1,248**	**0.3**
Nasopharynx	467	0.3	176	0.1	**643**	**0.2**
Tonsil	657	0.4	182	0.1	**839**	**0.2**
Oropharynx	663	0.4	243	0.1	**906**	**0.2**
Hypopharynx	260	0.2	64	0	**324**	**0.1**
Other oral cavity and pharynx	1,275	0.8	378	0.2	**1,653**	**0.4**
**Digestive system**	**85,255**	**52.9**	**63,528**	**31.4**	**148,783**	**41.1**
Esophagus	11,731	7.1	2,958	1.5	**14,689**	**4.0**
Stomach	6,793	4.3	4,468	2.3	**11,261**	**3.2**
Small intestine	705	0.5	565	0.3	**1,270**	**0.4**
Colon and rectum	27,230	17.3	24,583	12.1	**51,813**	**14.5**
Colon excluding rectum	21,494	13.8	20,469	10.1	**41,963**	**11.7**
Rectum and rectosigmoid junction	5,736	3.5	4,114	2.1	**9,850**	**2.7**
Anus, anal canal, and anorectum	371	0.2	529	0.3	**900**	**0.2**
Liver and intrahepatic bile duct	16,300	9.5	7,732	3.8	**24,032**	**6.5**
Gallbladder	750	0.5	1,410	0.7	**2,160**	**0.6**
Other biliary	704	0.5	814	0.4	**1,518**	**0.4**
Pancreas	19,854	12.4	19,142	9.4	**38,996**	**10.8**
Retroperitoneum	111	0.1	82	0	**193**	**0.1**
Peritoneum, omentum, and mesentery	71	0	679	0.3	**750**	**0.2**
Other digestive organs	635	0.4	566	0.3	**1,201**	**0.3**
**Respiratory system**	**89,108**	**56.0**	**71,536**	**35.9**	**160,644**	**44.6**
Nose, nasal cavity, and middle ear	269	0.2	174	0.1	**443**	**0.1**
Larynx	2,994	1.8	735	0.4	**3,729**	**1.0**
Lung and bronchus	85,658	53.9	70,518	35.4	**156,176**	**43.4**
Pleura	42	0	24	0	**66**	**0**
Trachea, mediastinum, and other respiratory organs	145	0.1	85	0	**230**	**0.1**
**Bones and joints**	**832**	**0.5**	**621**	**0.3**	**1,453**	**0.4**
**Soft tissue including heart**	**2,378**	**1.5**	**2,182**	**1.2**	**4,560**	**1.3**
**Skin excluding basal and squamous**	**8,723**	**5.6**	**4,025**	**2.0**	**12,748**	**3.6**
Melanoma of the skin	6,239	4.0	3,155	1.6	**9,394**	**2.7**
Other nonepithelial skin	2,484	1.6	870	0.4	**3,354**	**0.9**
**Male and female breast**	**NA**	**NA**	**NA**	**NA**	**41,324**	**11.5**
Female breast	NA	NA	40,860	20.7	**NA**	**NA**
Male breast	464	0.3	NA	NA	**NA**	**NA**
**Female genital system**	**NA**	**NA**	**29,828**	**15.2**	**NA**	**NA**
Cervix	NA	NA	4,217	2.3	**NA**	**NA**
Corpus and uterus, NOS	NA	NA	9,325	4.6	**NA**	**NA**
Corpus	NA	NA	3,903	1.9	**NA**	**NA**
Uterus, NOS	NA	NA	5,422	2.7	**NA**	**NA**
Ovary	NA	NA	14,276	7.2	**NA**	**NA**
Vagina	NA	NA	437	0.2	**NA**	**NA**
Vulva	NA	NA	1,003	0.5	**NA**	**NA**
Other female genital organs	NA	NA	570	0.3	**NA**	**NA**
**Male genital system**	**28,390**	**19.7**	**NA**	**NA**	**NA**	**NA**
Prostate	27,681	19.2	NA	NA	**NA**	**NA**
Testis	383	0.2	NA	NA	**NA**	**NA**
Penis	270	0.2	NA	NA	**NA**	**NA**
Other male genital organs	56	0	NA	NA	**NA**	**NA**
**Urinary system**	**20,765**	**13.6**	**9,747**	**4.8**	**30,512**	**8.5**
Urinary bladder	11,294	7.7	4,463	2.1	**15,757**	**4.4**
Kidney and renal pelvis	8,967	5.6	4,939	2.5	**13,906**	**3.9**
Ureter	251	0.2	183	0.1	**434**	**0.1**
Other urinary organs	253	0.2	162	0.1	**415**	**0.1**
**Eye and orbit**	**168**	**0.1**	**151**	**0.1**	**319**	**0.1**
**Brain and other nervous system**	**8,491**	**5.2**	**6,852**	**3.6**	**15,343**	**4.3**
**Endocrine system**	**1,322**	**0.8**	**1,457**	**0.8**	**2,779**	**0.8**
Thyroid	838	0.5	1,012	0.5	**1,850**	**0.5**
Other endocrine including thymus	484	0.3	445	0.3	**929**	**0.3**
**Lymphomas**	**11,801**	**7.8**	**9,402**	**4.7**	**21,203**	**6.0**
Hodgkin lymphoma	633	0.4	457	0.2	**1,090**	**0.3**
Non-Hodgkin lymphoma	11,168	7.4	8,945	4.4	**20,113**	**5.7**
**Myeloma**	**6,407**	**4.2**	**5,394**	**2.7**	**11,801**	**3.3**
**Leukemias**	**13,625**	**9.1**	**9,924**	**5.0**	**23,549**	**6.7**
Acute lymphocytic leukemia	800	0.5	625	0.4	**1,425**	**0.4**
Chronic lymphocytic leukemia	2,786	1.9	1,871	0.9	**4,657**	**1.3**
Acute myeloid leukemia	5,590	3.7	4,121	2.1	**9,711**	**2.8**
Chronic myeloid leukemia	578	0.4	411	0.2	**989**	**0.3**
Other leukemias	3,871	2.6	2,896	1.4	**6,767**	**1.9**
**Mesothelioma**	**1,911**	**1.3**	**586**	**0.3**	**2,497**	**0.7**
**Miscellaneous**	**21,647**	**13.8**	**18,583**	**9.1**	**40,230**	**11.2**

**Abbreviations:** NA = not applicable; NOS = not otherwise specified.

*Rates are the number of deaths per 100,000 persons and are age-adjusted to the 2000 U.S. standard population (19 age groups – Census P25–1130). For more information, see USCS technical notes (https://www.cdc.gov/cancer/npcr/uscs/pdf/uscs-2013-technical-notes.pdf).

^†^Data are from the National Vital Statistics System (NVSS).

Four cancer sites accounted for 48% of all cases diagnosed in 2013, including 230,815 female breast cancers, 212,584 lung and bronchus cancers (111,907 among men and 100,677 among women), 176,450 prostate cancers, and 136,119 colon and rectum cancers (71,099 among men and 65,020 among women) (Table 1). These four sites also accounted for 47% of cancer deaths in 2013, including 156,176 lung cancer deaths, 51,813 colon and rectum cancer deaths, 40,860 female breast cancer deaths, and 27,681 prostate cancer deaths (Table 2).

By state, overall (all cancer sites combined) cancer incidence rates in 2013 ranged from 364 to 512 cases per 100,000 persons (Table 3), and overall cancer death rates ranged from 128 to 199 deaths per 100,000 persons (Table 4). The *Healthy People 2020* target (*5*) for overall cancer death rate (161.4 deaths per 100,000) has been reached in 21 states.

**TABLE 3:** Reported number and rate* of invasive^†^ cancer cases, all sites combined, by geographic division and area — United States, 2013^§^

Area/State	All races/ethnicities	
	No.	Rate
**Northeast**	**319,548**	**478.5**
**New England**	**82,466**	**464.8**
Connecticut	20,510	474.2
Maine	8,366	463.8
Massachusetts	36,097	457.5
New Hampshire	7,886	479.2
Rhode Island	6,097	479.4
Vermont	3,510	437.1
**Middle Atlantic**	**237,082**	**483.5**
New Jersey	49,960	483.1
New York	109,560	484.3
Pennsylvania	77,562	483.0
Midwest	350,820	448.1
**East North Central**	**242,838**	**448.2**
Illinois	64,959	454.9
Indiana	32,372	438.8
Michigan	52,067	440.1
Ohio	62,802	452.4
Wisconsin	30,638	451.1
**West North Central**	**107,982**	**447.7**
Iowa	16,911	456.1
Kansas	14,572	450.9
Minnesota	27,770	451.8
Missouri	31,628	442.6
Nebraska	9,176	437.6
North Dakota	3,508	433.6
South Dakota	4,417	450.1
**South**	**578,843**	**433.6**
**South Atlantic**	**316,742**	**432.0**
Delaware	5,681	502.0
District of Columbia	2,780	445.2
Florida	108,216	413.0
Georgia	45,984	450.3
Maryland	29,824	451.0
North Carolina	49,970	445.4
South Carolina	24,809	436.9
Virginia	38,151	418.5
West Virginia	11,327	464.0
**East South Central**	**101,032**	**464.8**
Alabama	25,340	444.0
Kentucky	26,068	511.7
Mississippi	15,482	459.9
Tennessee	34,142	450.9
**West South Central**	**161,069**	**419.0**
Arkansas	15,879	454.0
Louisiana	24,184	476.3
Oklahoma	19,044	440.3
Texas	101,962	399.4
**West**	—^¶^	—
**Mountain**	—	—
Arizona	28,418	370.6
Colorado	21,764	396.1
Idaho	7,358	419.5
Montana	5,610	437.0
Nevada	—	—
New Mexico	8,728	363.7
Utah	9,626	393.2
Wyoming	2,517	382.0
**Pacific**	**225,898**	**412.3**
Alaska	2,664	410.4
California	160,911	402.8
Hawaii	7,000	419.8
Oregon	20,458	431.5
Washington	34,865	450.3

*Rates are the number of cases per 100,000 persons and are age-adjusted to the 2000 U.S. standard population (19 age groups – Census P25–1130). For more information, see USCS technical notes (https://www.cdc.gov/cancer/npcr/uscs/pdf/uscs-2013-technical-notes.pdf).

^†^Invasive cancer excludes basal and squamous cell carcinomas of the skin except when these occur on the skin of the genital organs, and in situ cancers except urinary bladder.

^§^Data are compiled from cancer registries that meet the data quality criteria for all invasive cancer sites combined (all registries except Nevada, covering approximately 99% of the U.S. population).

^¶^Rates and counts are not presented for the West Census Region, the Mountain Census Division, or Nevada because data from Nevada are not included in this analysis.

**TABLE 4:** Reported cancer deaths and death rates,* all sites combined, by geographic division and area — United States, 2013^†^

Area/State	All races/ethnicities	
	No.	Rate
**United States**	**584,872**	**163.0**
**Northeast**	**109,494**	**160.1**
**New England**	**28,932**	**159.4**
Connecticut	6,619	147.8
Maine	3,227	174.8
Massachusetts	12,858	159.7
New Hampshire	2,584	158.6
Rhode Island	2,326	173.9
Vermont	1,318	164.1
**Middle Atlantic**	**80,562**	**160.4**
New Jersey	16,315	156.0
New York	35,735	155.5
Pennsylvania	28,512	170.0
Midwest	135,293	170.7
**East North Central**	**94,527**	**173.0**
Illinois	24,491	171.7
Indiana	13,258	179.4
Michigan	20,367	170.2
Ohio	24,986	177.4
Wisconsin	11,425	164.6
**West North Central**	**40,766**	**165.6**
Iowa	6,509	168.2
Kansas	5,379	162.9
Minnesota	9,601	155.1
Missouri	12,955	179.1
Nebraska	3,459	160.7
North Dakota	1,286	150.8
South Dakota	1,577	154.1
**South**	**223,673**	**168.4**
**South Atlantic**	**120,225**	**162.8**
Delaware	1,905	167.1
District of Columbia	1,095	177.7
Florida	42,734	154.9
Georgia	16,417	168.1
Maryland	10,608	163.0
North Carolina	18,589	167.7
South Carolina	9,745	174.0
Virginia	14,414	162.3
West Virginia	4,718	190.5
**East South Central**	**40,890**	**189.5**
Alabama	10,328	182.1
Kentucky	10,082	199.3
Mississippi	6,527	196.5
Tennessee	13,953	185.4
**West South Central**	**62,558**	**167.3**
Arkansas	6,688	189.6
Louisiana	9,419	188.7
Oklahoma	8,039	185.4
Texas	38,412	156.9
**West**	**116,412**	**148.3**
**Mountain**	**35,623**	**146.1**
Arizona	11,347	146.4
Colorado	7,357	139.2
Idaho	2,707	156.3
Montana	1,997	154.0
Nevada	4,817	164.9
New Mexico	3,481	145.1
Utah	2,971	127.9
Wyoming	946	147.7
**Pacific**	**80,789**	**149.3**
Alaska	1,016	173.1
California	57,714	146.6
Hawaii	2,332	134.9
Oregon	7,799	163.2
Washington	11,928	156.3

*Rates are the number of deaths per 100,000 persons and are age-adjusted to the 2000 U.S. standard population (19 age groups – Census P25–1130). For more information, see USCS technical notes (https://www.cdc.gov/cancer/npcr/uscs/pdf/uscs-2013-technical-notes.pdf).

^†^Data are from the National Vital Statistics System (NVSS).

Cancer incidence (Table 5) and death (Table 6) rates increase with age. In 2013, among persons in the youngest age group (<15 years), 10,172 new cancer cases (rate: 17 cases per 100,000 persons) and 1,287 cancer deaths (rate: two deaths per 100,000 persons) were reported. Among persons aged ≥65 years, 851,505 new cancer cases (rate: 1,920 cases per 100,000 persons) and 407,558 cancer deaths (rate: 911 deaths per 100,000 persons) were reported. Overall, 55% of cancer cases and 70% of cancer deaths in 2013 occurred among persons aged ≥65 years.

**TABLE 5:** Reported number and rate* of invasive^†^ cancer cases, by primary cancer site and age group — United States, 2013^§^

Cancer site	Age group (yrs)										Total (all ages)	
	<15		15–24		25–39		40–64		≥65			
	No.	Rate	No.	Rate	No.	Rate	No.	Rate	No.	Rate	No.	Rate (crude)
**All sites combined**	**10,172**	**16.8**	**12,482**	**28.6**	**59,280**	**95.7**	**625,691**	**606.5**	**851,505**	**1,920.3**	**1,559,130**	**497.0**
Oral cavity and pharynx	107	0.2	226	0.5	1,138	1.8	21,699	21.0	18,547	41.8	**41,717**	**13.3**
Esophagus	—^¶^	—	—	—	131	0.2	6,513	6.3	9,782	22.1	**16,432**	**5.2**
Stomach	—	—	67	0.2	639	1.0	8,301	8.0	14,137	31.9	**—**	**7.4**
Colon and rectum	48	0.1	359	0.8	3,463	5.6	52,994	51.4	79,255	178.7	**136,119**	**43.4**
Liver and intrahepatic bile duct	180	0.3	76	0.2	307	0.5	15,104	14.6	13,806	31.1	**29,473**	**9.4**
Pancreas	—	—	57	0.1	446	0.7	14,506	14.1	29,361	66.2	**—**	**14.1**
Larynx	—	—	—	—	93	0.2	5,998	5.8	6,337	14.3	**12,442**	**4.0**
Lung and bronchus	35	0.1	101	0.2	948	1.5	65,462	63.5	146,038	329.3	**212,584**	**67.8**
Melanomas of the skin	120	0.2	912	2.1	5,477	8.8	29,698	28.8	35,736	80.6	**71,943**	**22.9**
Female breast	—	—	189	0.9	9,965	32.3	117,033	222.1	103,622	415.9	**—**	**144.8**
Cervix	—	—	120	0.6	2,796	9.1	6,605	12.5	2,428	9.7	**—**	**7.5**
Corpus and uterus, NOS	—	—	62	0.3	1,607	5.2	26,844	51.0	22,044	88.5	**—**	**31.7**
Ovary	115	0.4	346	1.6	1,028	3.3	9,498	18.0	9,940	39.9	**20,927**	**13.1**
Prostate	—	—	—	—	94	0.3	74,866	148.3	101,478	522.4	**176,450**	**114.3**
Testis	69	0.2	1,464	6.5	4,222	13.6	2,396	4.7	200	1.0	**8,351**	**5.4**
Urinary bladder	20	0	55	0.1	513	0.8	17,787	17.2	52,576	118.6	**70,951**	**22.6**
Kidney and renal pelvis	549	0.9	161	0.4	2,140	3.5	25,809	25.0	28,289	63.8	**56,948**	**18.2**
Brain and nervous system	2,170	3.6	954	2.2	2,023	3.3	8,381	8.1	8,320	18.8	**21,848**	**7.0**
Thyroid	222	0.4	1,936	4.4	9,820	15.8	25,477	24.7	10,238	23.1	**47,693**	**15.2**
Hodgkin lymphoma	327	0.5	1,516	3.5	2,183	3.5	2,631	2.6	1,576	3.6	**8,233**	**2.6**
Non-Hodgkin lymphoma	591	1.0	923	2.1	2,909	4.7	23,046	22.3	37,253	84.0	**64,722**	**20.6**
Myeloma	—	—	—	—	278	0.4	7,953	7.7	14,313	32.3	**22,562**	**7.2**
Leukemias	2,896	4.8	1,241	2.8	2,162	3.5	13,922	13.5	25,139	56.7	**45,360**	**14.5**
Mesothelioma	—	—	—	—	46	0.1	676	0.7	2,478	5.6	**3,207**	**1.0**
Kaposi Sarcoma	—	—	43	0.1	300	0.5	460	0.4	301	0.7	**—**	**0.4**

**Abbreviation:** NOS = not otherwise specified.

*Rates are the number of cases per 100,000 persons. For more information, see USCS technical notes (https://www.cdc.gov/cancer/npcr/uscs/pdf/uscs-2013-technical-notes.pdf).

^†^Invasive cancer excludes basal and squamous cell carcinomas of the skin except when these occur on the skin of the genital organs, and in situ cancers except urinary bladder. Urinary bladder cancer includes invasive and in situ.

^§^Data are compiled from cancer registries that meet the data quality criteria for all invasive cancer sites combined (all registries except Nevada, covering approximately 99% of the U.S. population).

^¶^Counts and rates are suppressed if <16 cases were reported in a specific category. Some counts and rates are suppressed as complementary cell suppression.

**TABLE 6:** Reported number and rate* of cancer deaths, by primary cancer site and age group — United States, 2013^†^

Cancer site	Age group (yrs)										Total (all ages)	
	<15		15–24		25–39		40–64		≥65			
	No.	Rate	No.	Rate	No.	Rate	No.	Rate	No.	Rate	No.	Rate (crude)
**All sites combined**	**1,287**	**2.1**	**1,496**	**3.4**	**7,446**	**11.9**	**167,085**	**160.5**	**407,558**	**911.3**	**584,872**	**184.8**
Oral cavity and pharynx	—^§^	—	—	—	114	0.2	3,608	3.5	5,111	11.4	**8,850**	**2.8**
Esophagus	—	—	—	—	87	0.1	5,161	5.0	9,441	21.1	**14,689**	**4.6**
Stomach	—	—	19	0	287	0.5	3,504	3.4	7,450	16.7	—	**3.6**
Colon and rectum	—	—	46	0.1	805	1.3	15,356	14.8	35,603	79.6	—	**16.4**
Liver and intrahepatic bile duct	33	0.1	32	0.1	211	0.3	10,089	9.7	13,667	30.6	**24,032**	**7.6**
Pancreas	—	—	—	—	183	0.3	10,756	10.3	28,045	62.7	**38,996**	**12.3**
Larynx	—	—	—	—	—	—	1,350	1.3	2,372	5.3	**3,729**	**1.2**
Lung and bronchus	—	—	22	0	383	0.6	42,733	41.1	113,032	252.7	—	**49.3**
Melanomas of the skin	—	—	26	0.1	352	0.6	3,082	3.0	5,932	13.3	—	**3.0**
Female breast	—	—	—	—	961	3.1	15,774	29.7	24,114	96.0	**40,860**	**25.4**
Cervix	—	—	—	—	422	1.4	2,369	4.5	1,413	5.6	**4,217**	**2.6**
Corpus and uterus, NOS	—	—	—	—	106	0.3	3,024	5.7	6,194	24.7	**9,325**	**5.8**
Ovary	—	—	32	0.1	170	0.5	4,614	8.7	9,458	37.7	—	**8.9**
Prostate	—	—	—	—	—	—	3,033	6.0	24,638	125.7	**27,681**	**17.8**
Testis	—	—	32	0.1	130	0.4	167	0.3	53	0.3	—	**0.2**
Urinary bladder	—	—	—	—	31	0	2,450	2.4	13,275	29.7	**15,757**	**5.0**
Kidney and renal pelvis	40	0.1	19	0	138	0.2	4,256	4.1	9,453	21.1	**13,906**	**4.4**
Brain and nervous system	437	0.7	241	0.5	751	1.2	6,080	5.8	7,834	17.5	**15,343**	**4.8**
Thyroid	—	—	—	—	17	0	473	0.5	1,357	3.0	**1,850**	**0.6**
Hodgkin lymphoma	—	—	32	0.1	137	0.2	334	0.3	583	1.3	—	**0.3**
Non-Hodgkin lymphoma	29	0	91	0.2	344	0.5	4,089	3.9	15,560	34.8	**20,113**	**6.4**
Myeloma	—	—	—	—	33	0.1	2,478	2.4	9,289	20.8	**11,801**	**3.7**
Leukemias	350	0.6	345	0.8	643	1.0	4,574	4.4	17,637	39.4	**23,549**	**7.4**
Mesothelioma	—	—	—	—	—	—	443	0.4	2,045	4.6	**2,497**	**0.8**

**Abbreviation:** NOS = not otherwise specified.

*Rates are the number of deaths per 100,000 persons. For more information, see USCS technical notes (https://www.cdc.gov/cancer/npcr/uscs/pdf/uscs-2013-technical-notes.pdf).

^†^Data are from the National Vital Statistics System (NVSS).

^§^Counts and rates are suppressed if <16 cases were reported in a specific category. Some counts and rates are suppressed as complementary cell suppression.

**FIGURE 1:**
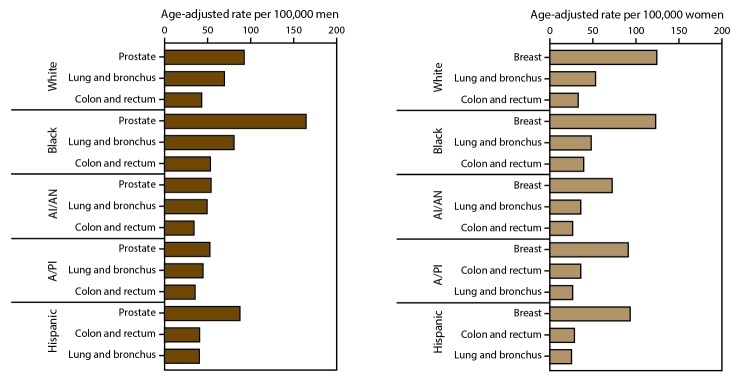
Age-adjusted rate* of invasive^†^ cancer cases for most common sites, by sex and race/ethnicity^§^ — United States, 2013^¶^ **Abbreviations**: AI/AN = American Indian/Alaska Native; A/PI = Asian/Pacific Islander. **Sources**: CDC’s National Program of Cancer Registries and National Cancer Institute’s Surveillance, Epidemiology, and End Results program. * Rates are the number of cases per 100,000 persons and are age-adjusted to the 2000 U.S. standard population (19 age groups – Census P25–1130). For more information, see USCS technical notes (https://www.cdc.gov/cancer/npcr/uscs/pdf/uscs-2013-technical-notes.pdf). ^†^ Invasive cancer excludes basal and squamous cell carcinomas of the skin except when these occur on the skin of the genital organs, and in situ cancers except urinary bladder. ^§^ Race categories are not mutually exclusive from Hispanic origin. The Hispanic category excludes any cases from Virginia because information about ethnicity was missing on 85% of cases. Rates are not presented for persons of unknown or other race. Data for specified racial or ethnic populations other than white and black should be interpreted with caution. For more information, see USCS technical notes (https://www.cdc.gov/cancer/npcr/uscs/pdf/uscs-2013-technical-notes.pdf#nameddest=IntRaceEthnicityData). ^¶^ Data are compiled from cancer registries that meet the data quality criteria for all invasive cancer sites combined (all registries except Nevada, covering approximately 99% of the U.S. population). Caution should be used when comparing incidence and death rates because of the difference in population coverage.

**FIGURE 2:**
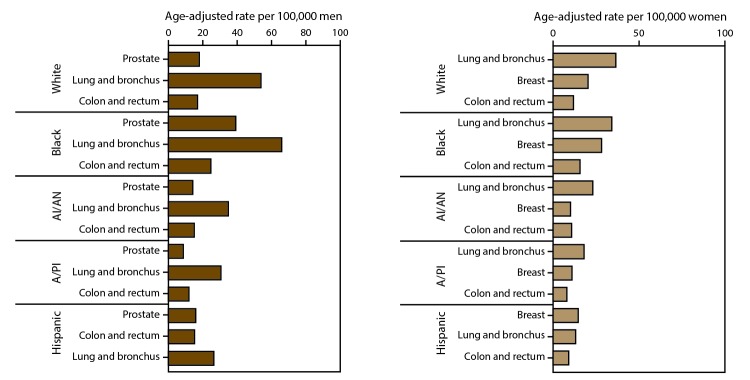
Age-adjusted rate* of cancer deaths for most common sites, by sex and race/ethnicity^†^ — United States, 2013^§^ **Abbreviations:** AI/AN = American Indian/Alaska Native; A/PI = Asian/Pacific Islander. * Rates are the number of cases per 100,000 persons and are age-adjusted to the 2000 U.S. standard population (19 age groups – Census P25–1130). For more information, see USCS technical notes (https://www.cdc.gov/cancer/npcr/uscs/pdf/uscs-2013-technical-notes.pdf). ^†^ Data are from the National Vital Statistics System (NVSS). Data for death rates cover 100% of the U.S. population. Use caution when comparing incidence and death rates because of potential differences in population coverage. ^§^ Race categories are not mutually exclusive from Hispanic origin. Rates are not presented for persons of unknown or other race. Data for specified racial or ethnic populations other than white and black should be interpreted with caution. For more information, see USCS technical notes (https://www.cdc.gov/cancer/npcr/uscs/pdf/uscs-2013-technical-notes.pdf#nameddest=IntRaceEthnicityData).

In 2013, by race, blacks had the highest cancer incidence (Table 7) and death (Table 8) rates while American Indians/Alaska Natives had the lowest cancer incidence and Asians/Pacific Islanders had the lowest cancer death rates. By ethnicity, overall and for most cancer sites, Hispanics had lower cancer incidence (Table 9) and death rates (Table 10) than did non-Hispanics. Differences in cancer incidence (Figure 1) and death (Figure 2) rates by race and ethnicity might reflect differences in risk factors, screening, and treatment although rates among some populations might be under- or overestimated because of misclassification of race or ethnicity.

**TABLE 7:** Reported number and rate* of invasive^†^ cancer cases, by primary cancer site and race^§^ — United States, 2013^¶^

Cancer site	AI/AN		A/PI		Black		White	
	No.	Rate	No.	Rate	No.	Rate	No.	Rate
**All sites combined**	**8,676**	**276.4**	**47,802**	**284.0**	**170,123**	**443.6**	**1,304,263**	**439.3**
**Oral cavity and pharynx**	**244**	**7.1**	**1,373**	**7.8**	**3,644**	**9.0**	**35,858**	**11.8**
Lip	—**	—	18	0.1	26	0.1	1,816	0.6
Tongue	69	1.9	302	1.8	813	2.0	11,402	3.7
Salivary gland	17	0.5	186	1.1	423	1.1	3,710	1.3
Floor of mouth	—	—	36	0.2	221	0.5	1,693	0.5
Gum and other mouth	24	0.8	216	1.3	502	1.3	4,590	1.5
Nasopharynx	16	0.5	411	2.2	268	0.7	1,090	0.4
Tonsil	46	1.2	114	0.6	632	1.5	7,073	2.3
Oropharynx	17	0.5	19	0.1	267	0.6	1,590	0.5
Hypopharynx	19	0.5	60	0.4	362	0.9	1,889	0.6
Other oral cavity and pharynx	—	—	—	—	130	0.3	1,005	0.3
**Digestive system**	**1,919**	**63.3**	**11,416**	**70.1**	**35,835**	**94.9**	**227,267**	**75.5**
Esophagus	93	3.2	326	2.0	1,604	4.2	14,289	4.7
Stomach	202	6.9	1,694	10.7	3,563	9.9	17,338	5.8
Small intestine	33	1.0	184	1.1	1,406	3.7	6,482	2.2
Colon and rectum	905	29.9	4,921	29.6	16,843	44.9	111,623	37.5
Colon excluding rectum	597	20.6	3,138	19.3	12,379	33.5	79,695	26.8
Rectum and rectosigmoid junction	308	9.3	1,783	10.3	4,464	11.4	31,928	10.8
Anus, anal canal, and anorectum	33	0.9	84	0.5	729	1.8	5,661	1.9
Liver and intrahepatic bile duct	312	9.3	2,037	12.3	4,422	10.4	22,350	7.1
Gallbladder	43	1.7	196	1.2	626	1.8	3,019	1.0
Other biliary	36	1.3	343	2.2	604	1.7	5,331	1.8
Pancreas	221	7.7	1,433	9.2	5,492	15.1	36,908	12.1
Retroperitoneum	—	—	59	0.3	146	0.4	1,011	0.4
Peritoneum, omentum, and mesentery	17	0.5	62	0.4	164	0.4	1,720	0.6
Other digestive organs	18	0.6	77	0.5	236	0.7	1,535	0.5
**Respiratory system**	**1,251**	**43.8**	**5,756**	**36.8**	**24,488**	**66.2**	**195,297**	**64.4**
Nose, nasal cavity, and middle ear	—	—	94	0.6	252	0.6	1,930	0.7
Larynx	58	1.8	177	1.1	1,672	4.2	10,402	3.4
Lung and bronchus	1,175	41.5	5,446	35.0	22,489	61.2	182,352	60.1
Pleura	—	—	—	—	—	—	92	0
Trachea, mediastinum, and other respiratory organs	—	—	37	0.2	68	0.2	521	0.2
**Bones and joints**	**27**	**0.7**	**99**	**0.5**	**333**	**0.8**	**2,466**	**1.0**
**Soft tissue including heart**	**80**	**2.4**	**414**	**2.4**	**1,283**	**3.3**	**9,058**	**3.2**
**Skin excluding basal and squamous**	**192**	**6.1**	**323**	**1.9**	**746**	**2.0**	**72,417**	**25.1**
Melanomas of the skin	161	5.0	213	1.3	362	1.0	67,291	23.3
Other nonepithelial skin	31	1.1	110	0.7	384	1.0	5,126	1.8
**Male and female breast**	**1,279**	**38.6**	**8,946**	**49.9**	**27,160**	**70.1**	**192,817**	**66.0**
Female breast	1,272	72.3	8,914	91.1	26,854	122.9	191,077	124.4
Male breast	—	—	32	0.4	306	1.9	1,740	1.3
**Female genital system**	**624**	**34.2**	**3,529**	**35.8**	**10,267**	**46.7**	**76,112**	**49.1**
Cervix	118	6.3	588	5.9	1,936	9.0	9,058	7.0
Corpus and uterus, NOS	319	17.3	1,848	18.5	5,546	24.8	42,116	26.2
Corpus	309	16.7	1,796	18.0	5,185	23.2	40,946	25.4
Uterus, NOS	—	—	52	0.5	361	1.6	1,170	0.7
Ovary	145	8.1	887	9.2	1,969	9.1	17,688	11.4
Vagina	—	—	40	0.4	189	0.9	1,017	0.6
Vulva	25	1.6	73	0.8	395	1.8	4,324	2.7
Other female genital organs	—	—	93	0.9	232	1.1	1,909	1.2
**Male genital system**	**855**	**58.3**	**4,079**	**55.0**	**28,677**	**166.9**	**145,637**	**100.1**
Prostate	762	54.0	3,853	52.5	28,208	164.4	136,604	92.5
Testis	80	3.3	178	1.8	285	1.4	7,598	6.4
Penis	—	—	26	0.4	144	0.9	1,133	0.9
Other male genital organs	—	—	22	0.3	40	0.2	302	0.2
**Urinary system**	**746**	**24.3**	**2,618**	**16.4**	**11,088**	**29.7**	**114,606**	**38.3**
Urinary bladder	232	8.7	1,247	8.3	4,040	11.6	64,236	21.3
Kidney and renal pelvis	500	15.0	1,274	7.5	6,864	17.6	47,630	16.1
Ureter	—	—	65	0.4	71	0.2	1,749	0.6
Other urinary organs	—	—	32	0.2	113	0.3	991	0.3
**Eye and orbit**	**—**	**—**	**56**	**0.3**	**112**	**0.3**	**2,537**	**0.9**
**Brain and other nervous system**	**112**	**3.0**	**622**	**3.6**	**1,639**	**4.0**	**19,211**	**7.0**
Brain	104	2.8	566	3.3	1,487	3.7	18,096	6.5
Cranial nerves other nervous system	—	—	56	0.3	152	0.4	1,115	0.4
**Endocrine system**	**315**	**8.3**	**2,944**	**15.7**	**4,140**	**10.2**	**41,563**	**15.8**
Thyroid	298	7.8	2,796	14.9	3,759	9.3	39,758	15.1
Other endocrine including thymus	17	0.5	148	0.9	381	0.9	1,805	0.7
**Lymphomas**	**345**	**10.9**	**2,262**	**13.6**	**6,408**	**16.4**	**62,525**	**21.6**
Hodgkin lymphoma	37	1.0	216	1.1	1,109	2.6	6,688	2.6
Non-Hodgkin lymphoma	308	9.9	2,046	12.4	5,299	13.8	55,837	19.0
**Myeloma**	**124**	**4.3**	**595**	**3.7**	**4,659**	**12.8**	**16,845**	**5.6**
**Leukemias**	**234**	**7.3**	**1,206**	**7.1**	**3,811**	**10.1**	**39,006**	**13.6**
Acute lymphocytic leukemia	41	0.9	205	1.1	385	0.9	3,896	1.6
Chronic lymphocytic leukemia	49	1.8	144	0.9	1,115	3.1	13,229	4.3
Acute myeloid leukemia	80	2.6	539	3.2	1,268	3.4	12,307	4.3
Chronic myeloid leukemia	31	0.9	183	1.0	599	1.5	5,013	1.8
Other leukemias	33	1.2	135	0.8	444	1.2	4,561	1.6
**Mesothelioma**	**—**	**—**	**46**	**0.3**	**146**	**0.4**	**2,982**	**1.0**
**Kaposi Sarcoma**	**—**	**—**	**29**	**0.2**	**292**	**0.7**	**695**	**0.3**
**Miscellaneous**	**298**	**10.7**	**1,489**	**9.8**	**5,395**	**15.2**	**47,364**	**15.8**

**Abbreviations:** AI/AN = American Indian/Alaska Native; A/PI = Asian/Pacific Islander; NOS = not otherwise specified.

*Rates are the number of cases per 100,000 persons and are age-adjusted to the 2000 U.S. standard population (19 age groups – Census P25–1130). For more information, see USCS technical notes (https://www.cdc.gov/cancer/npcr/uscs/pdf/uscs-2013-technical-notes.pdf).

^†^Invasive cancer excludes basal and squamous cell carcinomas of the skin except when these occur on the skin of the genital organs, and in situ cancers except urinary bladder. Urinary bladder cancer includes invasive and in situ.

^§^Rates are not presented for persons of unknown or other race; therefore, categories do not sum to total. Data for specified racial populations other than white and black should be interpreted with caution. For more information, see USCS technical notes (https://www.cdc.gov/cancer/npcr/uscs/pdf/uscs-2013-technical-notes.pdf#nameddest=IntRaceEthnicityData).

**Counts and rates are suppressed if <16 cases were reported.

**TABLE 8:** Reported number and rate* of cancer deaths, by primary site and race^†^— United States, 2013^§^

Cancer site	AI/AN		A/I		Black		White	
	No.	Rate	No.	Rate	No.	Rate	No.	Rate
**All sites combined**	**3,109**	**111.2**	**15,703**	**100.0**	**67,952**	**189.5**	**498,108**	**163.3**
**Oral cavity and pharynx**	**32**	**1.0**	**314**	**1.9**	**1,076**	**2.8**	**7,428**	**2.4**
Lip	—^¶^	—	—	—	—	—	55	0
Tongue	—	—	53	0.3	203	0.5	1,945	0.6
Salivary gland	—	—	22	0.1	75	0.2	788	0.3
Floor of mouth	—	—	—	—	—	—	74	0
Gum and other mouth	—	—	46	0.3	122	0.3	1,077	0.3
Nasopharynx	—	—	127	0.7	95	0.2	416	0.1
Tonsil	—	—	—	—	86	0.2	736	0.2
Oropharynx	—	—	18	0.1	139	0.4	746	0.2
Hypopharynx	—	—	—	—	70	0.2	246	0.1
Other oral cavity and pharynx	—	—	28	0.2	275	0.7	1,345	0.4
**Digestive system**	**1,020**	**35.3**	**5,608**	**35.5**	**19,240**	**53.0**	**122,915**	**40.0**
Esophagus	74	2.6	271	1.7	1,308	3.5	13,036	4.2
Stomach	96	3.5	808	5.1	1,964	5.6	8,393	2.8
Small intestine	—	—	29	0.2	219	0.6	1,017	0.3
Colon and rectum	351	12.6	1,557	9.7	6,854	19.3	43,051	14.1
Colon excluding rectum	271	10.0	1,215	7.7	5,772	16.3	34,705	11.4
Rectum and rectosigmoid junction	80	2.6	342	2.1	1,082	3.0	8,346	2.7
Anus, anal canal, and anorectum	—	—	22	0.1	104	0.3	769	0.3
Liver and intrahepatic bile duct	256	8.0	1,542	9.5	3,385	8.4	18,849	6.0
Gallbladder	19	0.8	120	0.8	329	0.9	1,692	0.6
Other biliary	—	—	58	0.4	137	0.4	1,313	0.4
Pancreas	188	6.8	1,124	7.5	4,737	13.4	32,947	10.7
Retroperitoneum	—	—	—	—	20	0.1	161	0.1
Peritoneum, omentum, and mesentery	—	—	19	0.1	48	0.1	679	0.2
Other digestive organs	—	—	46	0.3	135	0.4	1,008	0.3
**Respiratory system**	**777**	**28.8**	**3,622**	**23.8**	**17,584**	**48.8**	**138,661**	**45.3**
Nose, nasal cavity, and middle ear	—	—	20	0.1	41	0.1	380	0.1
Larynx	19	0.6	53	0.3	599	1.6	3,058	1.0
Lung and bronchus	754	28.0	3,539	23.3	16,906	46.9	134,977	44.1
Pleura	—	—	—	—	—	—	60	0
Trachea, mediastinum, and other respiratory organs	—	—	—	—	35	0.1	186	0.1
**Bones and joints**	**—**	**—**	**41**	**0.2**	**164**	**0.4**	**1,238**	**0.4**
**Soft tissue including heart**	**30**	**0.9**	**156**	**0.9**	**552**	**1.4**	**3,822**	**1.3**
**Skin excluding basal and squamous**	**30**	**1.0**	**81**	**0.5**	**306**	**0.8**	**12,331**	**4.1**
Melanomas of the skin	16	0.5	46	0.3	141	0.4	9,191	3.1
Other nonepithelial skin	—	—	35	0.2	165	0.4	3,140	1.0
Male and female breast	166	5.5	1,059	6.2	6,167	16.6	33,932	11.2
Female breast	166	10.1	1,048	11.0	6,086	28.2	33,560	20.3
Male breast	—	—	—	—	60	0.4	340	0.3
**Female genital system**	**148**	**9.0**	**899**	**9.6**	**4,035**	**19.0**	**24,746**	**15.0**
Cervix	30	1.7	187	1.9	838	3.9	3,162	2.2
Corpus and uterus, NOS	37	2.2	263	2.8	1,764	8.3	7,261	4.3
Corpus	16	1.0	97	1.0	682	3.2	3,108	1.8
Uterus, NOS	21	1.3	166	1.8	1,082	5.1	4,153	2.5
Ovary	73	4.5	416	4.5	1,254	6.0	12,533	7.5
Vagina	—	—	—	—	54	0.3	373	0.2
Vulva	—	—	—	—	64	0.3	924	0.5
Other female genital organs	—	—	—	—	61	0.3	493	0.3
**Male genital system**	**138**	**14.5**	**492**	**8.9**	**4,594**	**39.5**	**23,166**	**18.4**
Prostate	131	14.2	473	8.6	4,528	39.1	22,549	18.0
Testis	—	—	—	—	—	—	354	0.3
Penis	—	—	—	—	42	0.3	218	0.2
Other male genital organs	—	—	—	—	—	—	45	0
**Urinary system**	**167**	**6.1**	**550**	**3.7**	**2,480**	**7.2**	**27,315**	**8.9**
Urinary bladder	48	1.7	248	1.8	1,167	3.5	14,294	4.7
Kidney and renal pelvis	118	4.3	272	1.7	1,260	3.5	12,256	4.0
Ureter	—	—	21	0.1	18	0.1	394	0.1
Other urinary organs	—	—	—	—	35	0.1	371	0.1
**Eye and orbit**	**—**	**—**	**—**	**—**	**—**	**—**	**310**	**0.1**
**Brain and other nervous system**	**68**	**2.1**	**376**	**2.2**	**941**	**2.4**	**13,958**	**4.7**
**Endocrine system**	**20**	**0.7**	**141**	**0.9**	**308**	**0.9**	**2,310**	**0.8**
Thyroid	17	0.6	98	0.7	182	0.5	1,553	0.5
Other endocrine including thymus	—	—	43	0.2	126	0.3	757	0.3
**Lymphomas**	**91**	**3.5**	**608**	**4.0**	**1,568**	**4.4**	**18,936**	**6.3**
Hodgkin lymphoma	—	—	21	0.1	108	0.3	958	0.3
Non-Hodgkin lymphoma	88	3.4	587	3.9	1,460	4.1	17,978	5.9
**Myeloma**	**63**	**2.3**	**214**	**1.4**	**2,157**	**6.4**	**9,367**	**3.1**
**Leukemias**	**104**	**3.7**	**580**	**3.7**	**1,987**	**5.7**	**20,878**	**7.0**
Acute lymphocytic leukemia	—	—	53	0.3	136	0.3	1,221	0.5
Chronic lymphocytic leukemia	—	—	33	0.2	378	1.1	4,236	1.4
Acute myeloid leukemia	48	1.7	317	2.0	778	2.2	8,568	2.9
Chronic myeloid leukemia	—	—	22	0.1	99	0.3	861	0.3
**Other leukemias**	24	1.0	155	1.0	596	1.7	5,992	2.0
**Mesothelioma**	**—**	**—**	**35**	**0.2**	**112**	**0.3**	**2,342**	**0.8**
**Miscellaneous**	**237**	**8.7**	**925**	**6.0**	**4,667**	**13.0**	**34,401**	**11.2**

**Abbreviations:** AI/AN = American Indian/Alaska Native; A/PI = Asian/Pacific Islander; NOS = not otherwise specified.

*Rates are the number of deaths per 100,000 persons and are age-adjusted to the 2000 U.S. standard population (19 age groups – Census P25–1130). For more information, see USCS technical notes (https://www.cdc.gov/cancer/npcr/uscs/pdf/uscs-2013-technical-notes.pdf).

^†^Data for specified racial populations other than white and black should be interpreted with caution. For more information, see USCS technical notes (https://www.cdc.gov/cancer/npcr/uscs/pdf/uscs-2013-technical-notes.pdf#nameddest=IntRaceEthnicityData).

^§^Data are from the National Vital Statistics System (NVSS).

^¶^Counts and rates are suppressed if <16 cases were reported.

**TABLE 9:** Reported number and rate* of invasive^†^ cancer cases, by primary cancer site and ethnicity^§^ — United States, 2013

Cancer site	Hispanic		Non-Hispanic	
	No.	Rate	No.	Rate
**All sites combined**	**117,332**	**343.7**	**1,403,357**	**450.8**
**Oral cavity and pharynx**	**2,360**	**6.8**	**38,279**	**12.1**
Lip	89	0.3	1,811	0.6
Tongue	653	1.9	11,797	3.7
Salivary gland	298	0.8	3,986	1.3
Floor of mouth	99	0.3	1,836	0.6
Gum and other mouth	346	1.1	4,942	1.6
Nasopharynx	152	0.4	1,607	0.5
Tonsil	438	1.2	7,317	2.3
Oropharynx	98	0.3	1,753	0.5
Hypopharynx	134	0.4	2,133	0.7
Other oral cavity and pharynx	53	0.2	1,097	0.3
**Digestive system**	**25,401**	**79.0**	**247,673**	**78.5**
Esophagus	800	2.6	15,228	4.7
Stomach	3,129	9.8	19,455	6.2
Small intestine	590	1.8	7,379	2.4
Colon and rectum	10,997	33.8	121,907	39.1
Colon excluding rectum	7,562	23.9	87,066	27.9
Rectum and rectosigmoid junction	3,435	10.0	34,841	11.2
Anus, anal canal, and anorectum	440	1.3	5,977	1.9
Liver and intrahepatic bile duct	4,311	12.9	24,545	7.4
Gallbladder	586	1.9	3,244	1.0
Other biliary	735	2.5	5,524	1.7
Pancreas	3,357	11.1	39,910	12.5
Retroperitoneum	141	0.4	1,055	0.4
Peritoneum, omentum, and mesentery	130	0.4	1,798	0.6
Other digestive organs	185	0.6	1,651	0.5
**Respiratory system**	**10,261**	**34.9**	**212,149**	**66.6**
Nose, nasal cavity, and middle ear	234	0.7	2,036	0.7
Larynx	809	2.5	11,310	3.5
Lung and bronchus	9,125	31.5	198,162	62.2
Pleura	—**	—	88	0
Trachea, mediastinum, and other respiratory organs	80	0.2	553	0.2
**Bones and joints**	**424**	**0.9**	**2,487**	**0.9**
**Soft tissue including heart**	1,210	3.0	9,571	3.3
**Skin excluding basal and squamous**	1,842	5.3	74,086	24.6
Melanomas of the skin	1,535	4.4	68,543	22.8
Other nonepithelial skin	307	0.9	5,543	1.8
**Male and female breast**	18,155	50.1	208,425	68.3
Female breast	18,041	93.4	206,497	127.4
Male breast	114	0.8	1,928	1.3
**Female genital system**	**9,034**	**45.4**	**80,637**	**49.0**
Cervix	2,010	9.3	9,689	7.0
Corpus and uterus, NOS	4,517	23.1	44,800	26.1
Corpus	4,301	21.9	43,444	25.3
Uterus, NOS	216	1.2	1,356	0.8
Ovary	1,912	9.7	18,507	11.3
Vagina	129	0.7	1,100	0.6
Vulva	295	1.7	4,492	2.7
Other female genital organs	171	0.9	2,049	1.2
**Male genital system**	**14,008**	**93.6**	**167,698**	**110.0**
Prostate	12,403	87.5	159,452	103.2
Testis	1,400	4.7	6,782	5.7
Penis	184	1.2	1,116	0.8
Other male genital organs	21	0.1	348	0.3
**Urinary system**	**8,926**	**27.7**	**119,098**	**38.0**
Urinary bladder	3,135	11.0	66,222	20.9
Kidney and renal pelvis	5,634	16.1	50,006	16.1
Ureter	96	0.4	1,760	0.6
Other urinary organs	61	0.2	1,110	0.4
**Eye and orbit**	**216**	**0.5**	**2,548**	**0.9**
**Brain and other nervous system**	**2,147**	**5.2**	**19,193**	**6.7**
Brain	1,966	4.8	18,049	6.3
Cranial nerves other nervous system	181	0.4	1,144	0.4
**Endocrine system**	**5,965**	**13.8**	**42,877**	**15.7**
Thyroid	5,700	13.2	40,801	14.9
Other endocrine including thymus	265	0.6	2,076	0.8
**Lymphomas**	**6,775**	**19.2**	**64,396**	**21.4**
Hodgkin lymphoma	1,084	2.4	6,934	2.7
Non-Hodgkin lymphoma	5,691	16.9	57,462	18.7
**Myeloma**	**1,908**	**6.1**	**20,128**	**6.4**
**Leukemias**	**4,243**	**10.7**	**40,185**	**13.4**
Acute lymphocytic leukemia	1,217	2.1	3,361	1.4
Chronic lymphocytic leukemia	621	2.1	14,188	4.4
Acute myeloid leukemia	1,321	3.6	12,690	4.2
Chronic myeloid leukemia	597	1.5	5,260	1.8
Other leukemias	487	1.4	4,686	1.6
**Mesothelioma**	**222**	**0.7**	**2,895**	**0.9**
**Kaposi Sarcoma**	**244**	**0.6**	**844**	**0.3**
**Miscellaneous**	**3,991**	**13.2**	**50,188**	**16.0**

**Abbreviation:** NOS = not otherwise specified.

*Rates are the number of cases per 100,000 persons and are age-adjusted to the 2000 U.S. standard population (19 age groups – Census P25–1130). For more information, see USCS technical notes (https://www.cdc.gov/cancer/npcr/uscs/pdf/uscs-2013-technical-notes.pdf).

^†^Invasive cancer excludes basal and squamous cell carcinomas of the skin except when these occur on the skin of the genital organs, and in situ cancers except urinary bladder. Urinary bladder cancer includes invasive and in situ.

^§^Rates and counts are not presented for persons of unknown ethnicity; therefore, categories do not sum to total. Data for specified ethnic populations should be interpreted with caution. For more information, see USCS technical notes (https://www.cdc.gov/cancer/npcr/uscs/pdf/uscs-2013-technical-notes.pdf#nameddest=IntRaceEthnicityData).

^¶^Data are compiled from cancer registries that meet the data quality criteria for all invasive cancer sites combined and have information on ethnicity (all registries except Nevada and Virginia, covering approximately 97% of the U.S. population). Data from Nevada did not meet the data quality criteria and information about ethnicity was missing on 85% of the cases from Virginia.

**Counts and rates are suppressed if <16 cases were reported. Some counts and rates are suppressed as complementary cell suppression.

**TABLE 10:** Reported number and rate* of cancer deaths, by primary cancer site and ethnicity^†^ — United States, 2013^§^

Cancer site	Hispanic		Non-Hispanic	
	No.	Rate	No.	Rate
**All sites combined**	**35,147**	**114.7**	**548,516**	**167.2**
**Oral cavity and pharynx**	**455**	**1.4**	**8,364**	**2.5**
Lip	—^¶^	—	58	0
Tongue	114	0.3	2,086	0.6
Salivary gland	39	0.1	845	0.3
Floor of mouth	—	—	81	0
Gum and other mouth	65	0.2	1,178	0.4
Nasopharynx	37	0.1	605	0.2
Tonsil	38	0.1	798	0.2
Oropharynx	58	0.2	843	0.3
Hypopharynx	23	0.1	298	0.1
Other oral cavity and pharynx	77	0.2	1,572	0.5
**Digestive system**	**11,914**	**38.7**	**136,551**	**41.3**
Esophagus	593	1.9	14,060	4.2
Stomach	1,633	5.1	9,605	3.0
Small intestine	78	0.3	1,190	0.4
Colon and rectum	3,568	11.7	48,133	14.7
Colon excluding rectum	2,902	9.6	38,973	11.9
Rectum and rectosigmoid junction	666	2.0	9,160	2.8
Anus, anal canal, and anorectum	50	0.1	846	0.3
Liver and intrahepatic bile duct	2,994	9.3	20,974	6.2
Gallbladder	254	0.9	1,905	0.6
Other biliary	123	0.4	1,393	0.4
Pancreas	2,473	8.4	36,458	11.0
Retroperitoneum	19	0.1	174	0.1
Peritoneum, omentum, and mesentery	40	0.1	706	0.2
Other digestive organs	89	0.3	1,107	0.3
**Respiratory system**	**5,629**	**19.7**	**154,680**	**46.8**
Nose, nasal cavity, and middle ear	33	0.1	408	0.1
Larynx	219	0.7	3,498	1.0
Lung and bronchus	5,353	18.8	150,502	45.6
Pleura	—	—	62	0
Trachea, mediastinum, and other respiratory organs	20	0.1	210	0.1
**Bones and joints**	**188**	**0.5**	**1,261**	**0.4**
**Soft tissue including heart**	**392**	**1.0**	**4,161**	**1.3**
**Skin excluding basal and squamous**	**357**	**1.2**	**12,372**	**3.8**
Melanomas of the skin	236	0.7	9,151	2.9
Other nonepithelial skin	121	0.4	3,221	1.0
**Male and female breast**	**2,695**	**8.0**	**38,540**	**11.8**
Female breast	2,671	14.5	38,104	21.3
Male breast	24	0.2	436	0.3
**Female genital system**	**2,280**	**12.6**	**27,492**	**15.4**
Cervix	521	2.5	3,682	2.3
Corpus and uterus, NOS	679	3.8	8,629	4.7
Corpus	222	1.2	3,673	2.0
Uterus, NOS	457	2.6	4,956	2.7
Ovary	942	5.3	13,312	7.4
Vagina	39	0.2	398	0.2
Vulva	63	0.4	939	0.5
Other female genital organs	36	0.2	532	0.3
**Male genital system**	**1,758**	**16.3**	**26,567**	**19.9**
Prostate	1,634	15.8	25,983	19.4
Testis	80	0.3	302	0.2
Penis	41	0.2	229	0.2
Other male genital organs	—	—	53	0
**Urinary system**	**1,720**	**5.9**	**28,730**	**8.7**
Urinary bladder	630	2.4	15,098	4.6
Kidney and renal pelvis	1,053	3.4	12,820	3.9
Ureter	18	0.1	416	0.1
Other urinary organs	19	0.1	396	0.1
**Eye and orbit**	**17**	**0.1**	**302**	**0.1**
**Brain and other nervous system**	**1,046**	**2.9**	**14,279**	**4.5**
**Endocrine system**	**275**	**0.8**	**2,498**	**0.8**
Thyroid	177	0.6	1,670	0.5
Other endocrine including thymus	98	0.2	828	0.3
**Lymphomas**	**1,593**	**5.3**	**19,571**	**6.1**
Hodgkin lymphoma	134	0.4	954	0.3
Non-Hodgkin lymphoma	1,459	4.9	18,617	5.7
Myeloma	774	2.7	11,003	3.4
**Leukemias**	**1,677**	**5.1**	**21,833**	**6.8**
Acute lymphocytic leukemia	320	0.7	1,103	0.4
Chronic lymphocytic leukemia	146	0.6	4,502	1.4
Acute myeloid leukemia	666	2.0	9,032	2.8
Chronic myeloid leukemia	70	0.2	916	0.3
Other leukemias	475	1.6	6,280	2.0
**Mesothelioma**	**125**	**0.4**	**2,369**	**0.7**
**Miscellaneous**	**2,238**	**7.5**	**37,898**	**11.5**

**Abbreviation:** NOS = not otherwise specified.

*Rates are the number of deaths per 100,000 persons and are age-adjusted to the 2000 U.S. standard population (19 age groups – Census P25–1130). For more information, see USCS technical notes (https://www.cdc.gov/cancer/npcr/uscs/pdf/uscs-2013-technical-notes.pdf).

^†^Data are from the National Vital Statistics System (NVSS).

^§^Rates are not presented for persons of unknown ethnicity; therefore, categories do not sum to total. Data for specified ethnic populations should be interpreted with caution. For more information, see USCS technical notes (https://www.cdc.gov/cancer/npcr/uscs/pdf/uscs-2013-technical-notes.pdf#nameddest=IntRaceEthnicityData).

^¶^Counts and rates are suppressed if <16 cases were reported.

**FIGURE 3:**
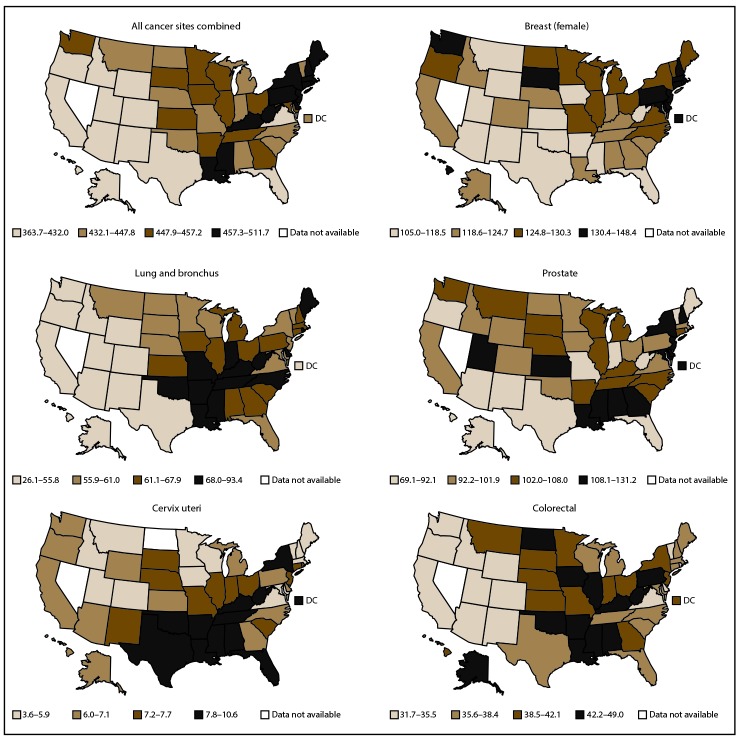
Age-adjusted rate* of invasive^†^ cancer cases, by primary cancer site and state — United States, 2013^§^ * Rates are the number of cases per 100,000 persons and are age-adjusted to the 2000 U.S. standard population (19 age groups – Census P25–1130). Rates are presented by quartiles. For more information, see USCS technical notes (https://www.cdc.gov/cancer/npcr/uscs/pdf/uscs-2013-technical-notes.pdf#nameddest=RegistriesPubCriteria). ^†^ Invasive cancer excludes basal and squamous cell carcinomas of the skin except when these occur on the skin of the genital organs, and in situ cancers except urinary bladder. ^§^ Data are compiled from cancer registries that meet the data quality criteria for all invasive cancer sites combined (all registries except Nevada, covering approximately 99% of the U.S. population).

**FIGURE 4:**
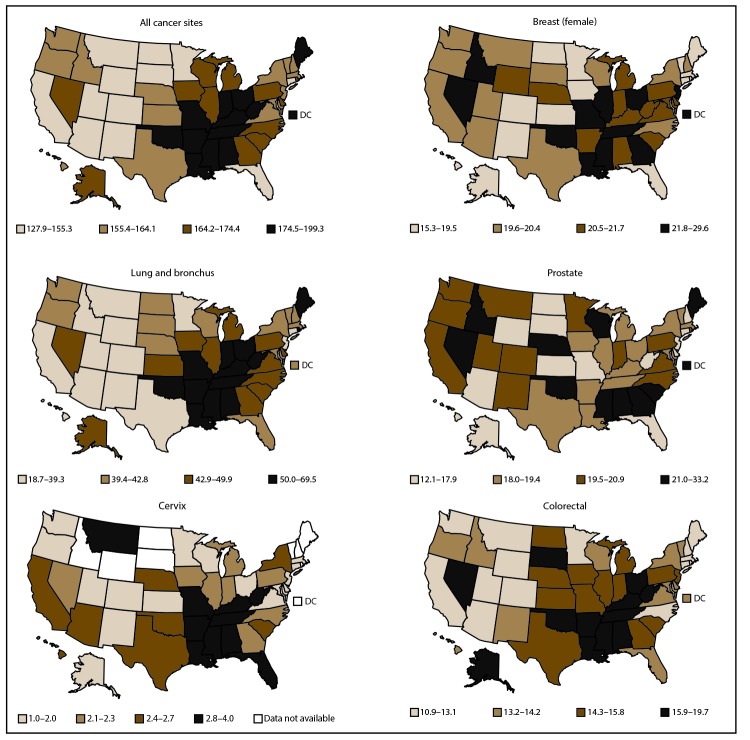
Age-adjusted rate* of cancer deaths, by primary cancer site and state — United States, 2013^†^ * Rates are the number of deaths per 100,000 persons and are age-adjusted to the 2000 U.S. standard population (19 age groups – Census P25–1130). Rates are presented by quartiles. For more information, see USCS technical notes (https://www.cdc.gov/cancer/npcr/uscs/pdf/uscs-2013-technical-notes.pdf#nameddest=RegistriesPubCriteria). ^†^ Data are from the National Vital Statistics System (NVSS). Data for death rates cover 100% of the U.S. population. Caution should be used when comparing incidence and death rates because of potential differences in population coverage.

By state and site, cancer incidence rates in 2013 ranged from 69 to 131 per 100,000 males for prostate cancer, from 105 to 148 per 100,000 females for breast cancer, from 4 to 11 per 100,000 females for cervical cancer, from 26 to 93 per 100,000 persons for lung cancer, and from 32 to 49 per 100,000 persons for colorectal cancer (Figure 3). By state and site, cancer death rates in 2013 ranged from 12 to 33 per 100,000 males for prostate cancer, from 15 to 30 per 100,000 females for breast cancer, from 1 to 4 per 100,000 females for cervical cancer, from 19 to 70 per 100,000 persons for lung cancer, and from 11 to 20 per 100,000 persons for colorectal cancer (Figure 4).

### Time Trends in Incidence and Death Rates

**FIGURE 5:**
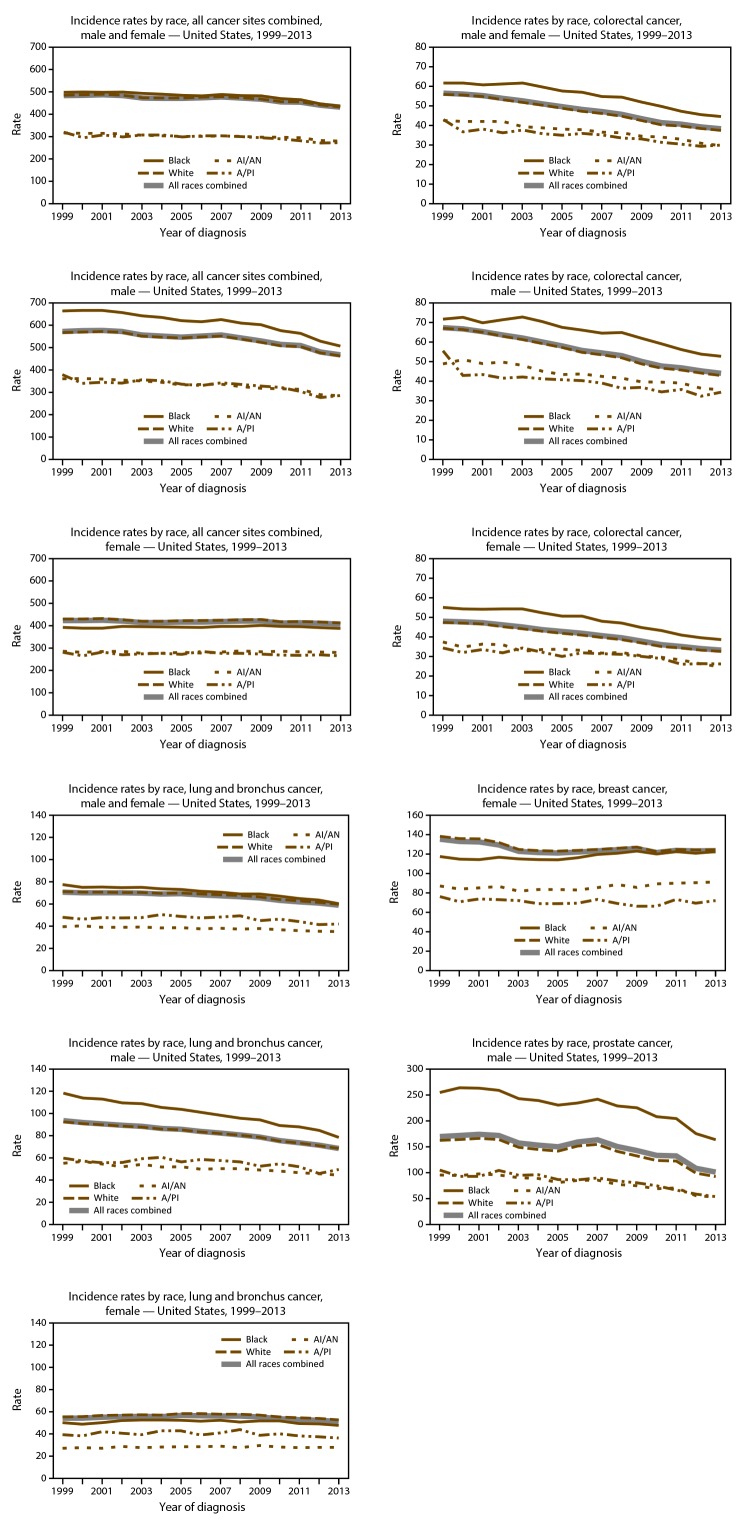
Age-adjusted rate* of invasive^†^ cancer cases, by primary cancer site, race,^§^ and sex — United States, 1999–2013^¶^ **Abbreviations:** AI/AN = American Indian/Alaska Native; A/PI = Asian/Pacific Islander. * Rates are the number of cases per 100,000 persons and are age-adjusted to the 2000 U.S. standard population (19 age groups – Census P25–1130). For more information, see USCS technical notes (https://www.cdc.gov/cancer/npcr/uscs/pdf/uscs-2013-technical-notes.pdf). ^†^ Invasive cancer excludes basal and squamous cell carcinomas of the skin except when these occur on the skin of the genital organs, and in situ cancers except urinary bladder. ^§^ Rates are not presented for persons of unknown or other race. Data for specified racial populations other than white and black should be interpreted with caution. For more information, see USCS technical notes (https://www.cdc.gov/cancer/npcr/uscs/pdf/uscs-2013-technical-notes.pdf#nameddest=IntRaceEthnicityData). ^¶^ Data are compiled from cancer registries that meet the data quality criteria for all invasive cancer sites combined for all years, 1999–2013 (all registries except Arkansas, District of Columbia, Mississippi, Nevada, South Dakota, Tennessee, and Virginia, covering approximately 92% of the U.S. population). See registry-specific data quality information for all years, 1999–2013 (https://www.cdc.gov/cancer/npcr/uscs/pdf/uscs-2013-technical-notes.pdf#nameddest=RegistriesPubCriteria). Caution should be used when comparing incidence and death rates because of potential differences in population coverage.

**FIGURE 6:**
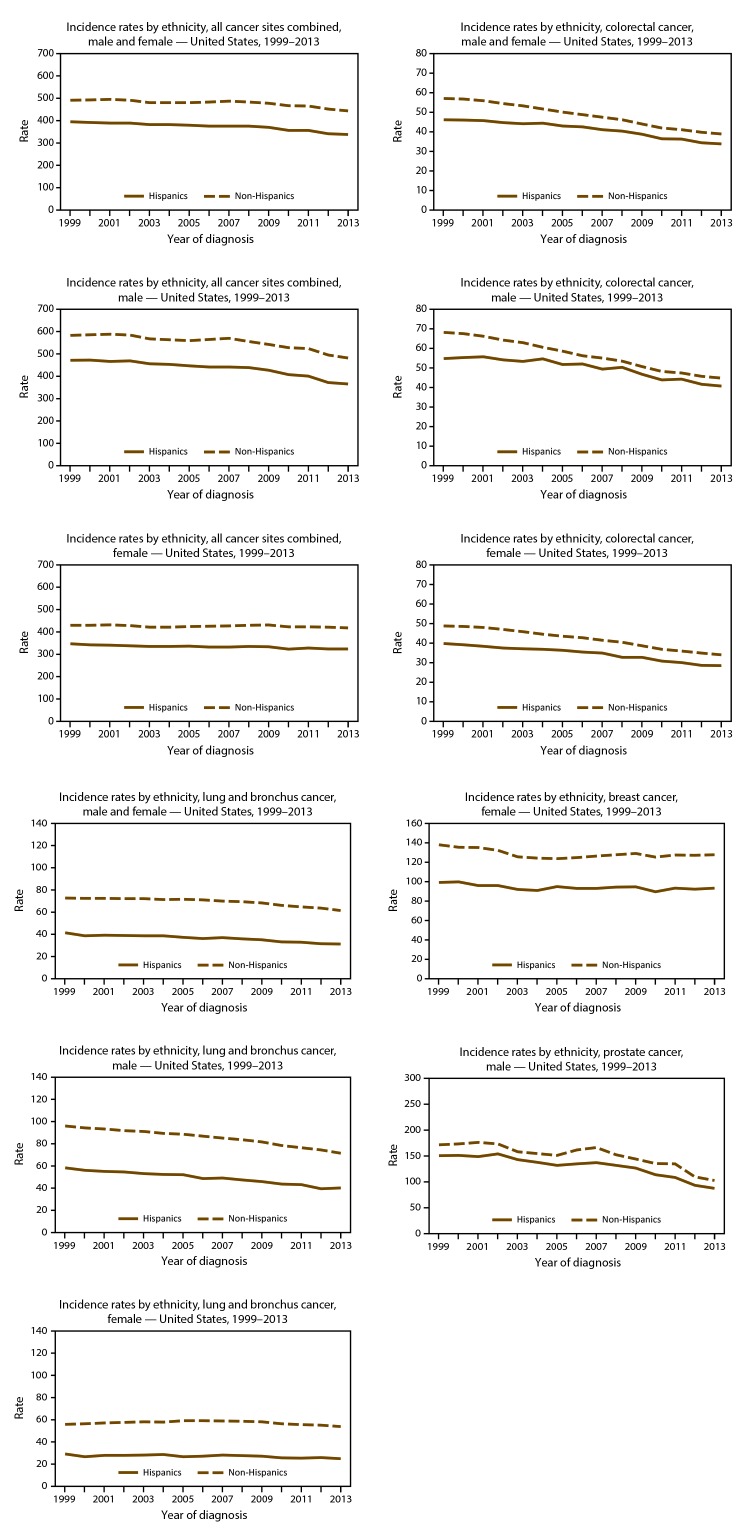
Age-adjusted rate* of invasive^†^ cancer cases, by primary cancer site, ethnicity,^§^ and sex — United States, 1999–2013^¶^ * Rates are the number of cases per 100,000 persons and are age-adjusted to the 2000 U.S. standard population (19 age groups – Census P25–1130). For more information, see USCS technical notes(https://www.cdc.gov/cancer/npcr/uscs/pdf/uscs-2013-technical-notes.pdf). ^†^ Invasive cancer excludes basal and squamous cell carcinomas of the skin except when these occur on the skin of the genital organs, and in situ cancers except urinary bladder. ^§^ Rates are not presented for persons of unknown ethnicity. Data for specified ethnical populations should be interpreted with caution. For more information, see USCS technical notes (https://www.cdc.gov/cancer/npcr/uscs/pdf/uscs-2013-technical-notes.pdf#nameddest=IntRaceEthnicityData). ^¶^ Data are compiled from cancer registries that meet the data quality criteria for all invasive cancer sites combined for all years, 1999–2013 (all registries except Arkansas, District of Columbia, Mississippi, Nevada, South Dakota, Tennessee, and Virginia, covering approximately 92% of the U.S. population). See registry-specific data quality information for all years, 1999–2013 (https://www.cdc.gov/cancer/npcr/uscs/pdf/uscs-2013-technical-notes.pdf#nameddest=RegistriesPubCriteria). Caution should be used when comparing incidence and death rates because of potential differences in population coverage.

**FIGURE 7:**
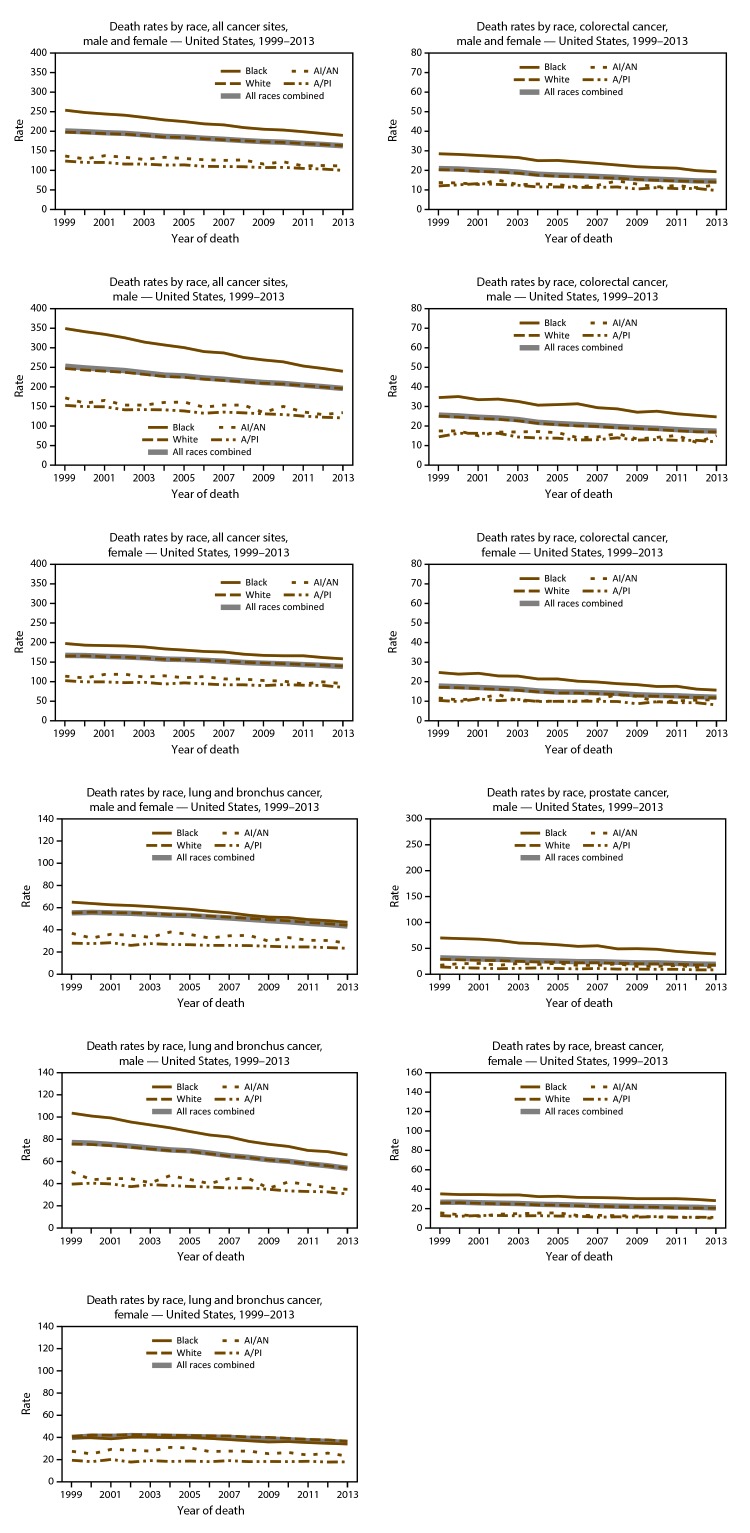
Age-adjusted rate* of cancer deaths, by primary cancer site, race,^†^ and sex — United States, 2013^§^ **Abbreviations:** AI/AN = American Indian/Alaska Native; A/PI = Asian/Pacific Islander. * Rates are the number of deaths per 100,000 persons and are age-adjusted to the 2000 U.S. standard population (19 age groups – Census P25–1130). For more information, see USCS technical notes (https://www.cdc.gov/cancer/npcr/uscs/pdf/uscs-2013-technical-notes.pdf). ^†^ Rates are not presented for persons of unknown or other race. Data for specified racial populations other than white and black should be interpreted with caution. For more information, see USCS technical notes (https://www.cdc.gov/cancer/npcr/uscs/pdf/uscs-2013-technical-notes.pdf#nameddest=IntRaceEthnicityData). ^§^ Data are from the National Vital Statistics System (NVSS). Data for death rates cover 100% of the U.S. population. Use caution when comparing incidence and death rates because of potential differences in population coverage.

**FIGURE 8:**
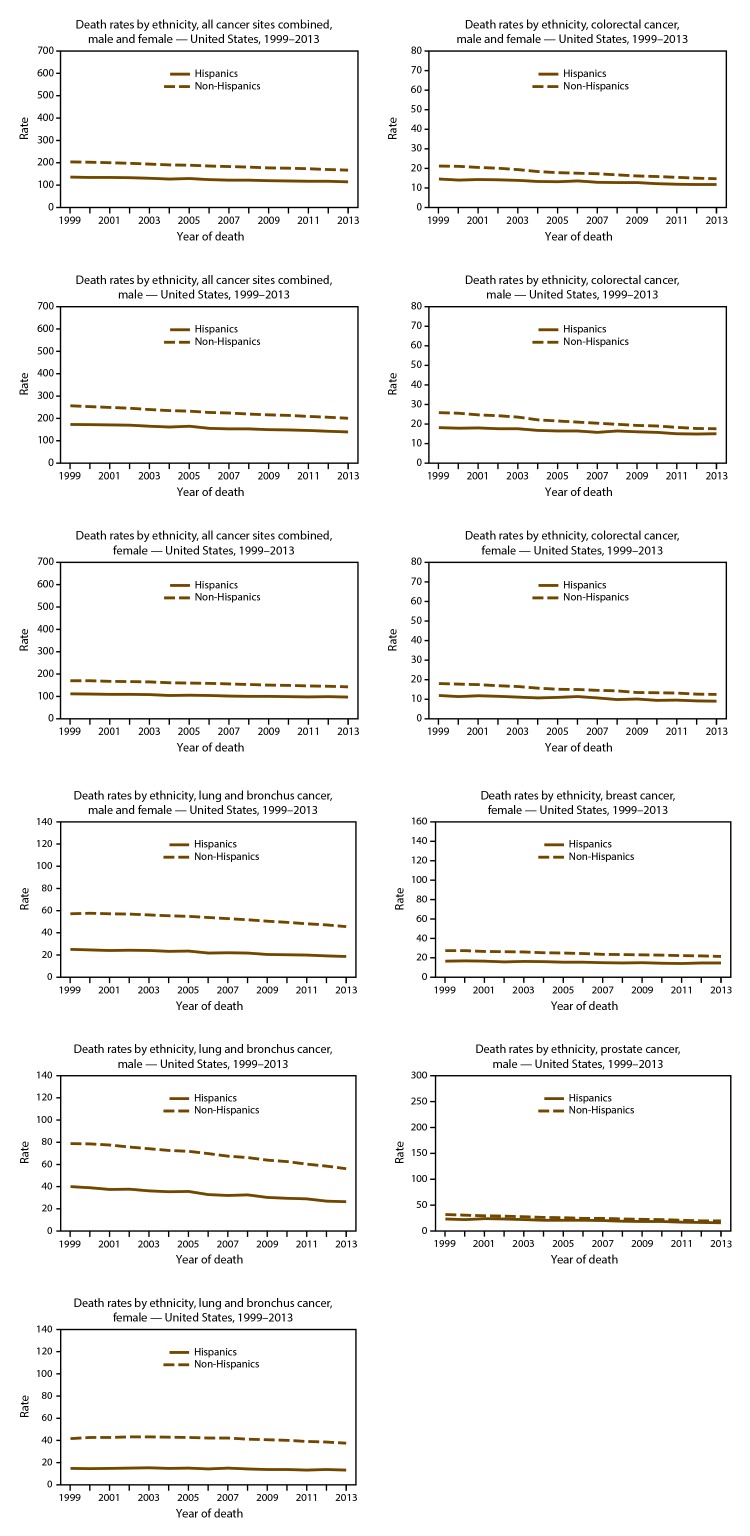
Reported rate* of cancer deaths, by primary cancer site, ethnicity,^†^ and sex — United States, 2013^§^ * Rates are the number of deaths per 100,000 persons and are age-adjusted to the 2000 U.S. standard population (19 age groups – Census P25–1130). For more information, see USCS technical notes (https://www.cdc.gov/cancer/npcr/uscs/pdf/uscs-2013-technical-notes.pdf). ^†^ Rates are not presented for persons of unknown ethnicity. Data for specified ethnic populations should be interpreted with caution. For more information, see USCS technical notes (https://www.cdc.gov/cancer/npcr/uscs/pdf/uscs-2013-technical-notes.pdf#nameddest=IntRaceEthnicityData). ^§^ Data are from the National Vital Statistics System (NVSS). Data for death rates cover 100% of the U.S. population. Use caution when comparing incidence and death rates because of potential differences in population coverage.

On the basis of data from registries meeting data quality criteria during 2004–2013, cancer incidence counts (Table 11) and rates (Table 12) are presented by state and year. Time trends in cancer incidence rates are presented by cancer site, sex, and race (Figure 5) and by cancer site, sex, and ethnicity (Figure 6). Cancer incidence rates declined from 484 cancer cases per 100,000 population in 1999 to 432 cases in 2013. Although lung cancer incidence declined steadily among men from 1999 to 2013, it increased among women from 1999 to 2005 and has since declined from 2005 to 2013. Prostate cancer incidence declined from 170 cases per 100,000 men in 1999 to 101 cases in 2013. Colorectal cancer incidence declined from 56 cases per 100,000 persons in 1999 to 38 cases in 2013. Female breast cancer incidence declined from 135 cases per 100,000 women in 1999 to 121 cases in 2005, increased to 126 cases in 2009, and declined again to 124 cases in 2013. Time trends in cancer death rates are presented by cancer site, sex, and race (Figure 7) and by cancer site, sex, and ethnicity (Figure 8). During 1999–2013, cancer death rates declined from 201 deaths per 100,000 persons in 1999 to 163 deaths in 2013; during the same period, death rates declined for each of the four most common cancers (Figure 5). Cancer death counts (Table 13) and rates (Table 14) are presented by state and year.

**TABLE 11:** Reported number of invasive* cancer cases, all cancer sites combined, by state — United States, 2004–2013^†^

State	2004	2005	2006	2007	2008	2009	2010	2011	2012	2013
Alaska	2,430	2,371	2,496	2,513	2,671	2,647	2,746	2,666	2,612	2,664
Alabama	22,251	22,665	23,351	24,215	25,316	25,307	25,305	25,195	25,383	25,340
Arkansas	13,918	14,203	14,417	14,871	14,833	14,984	15,190	15,151	15,512	15,879
Arizona	24,196	24,629	25,784	27,018	26,803	28,295	27,622	28,743	28,545	28,418
California	146,314	148,143	149,651	156,072	158,341	160,282	161,351	161,217	160,817	160,911
Colorado	18,345	18,882	19,261	20,696	20,948	21,627	21,448	21,761	21,846	21,764
Connecticut	19,474	19,775	20,534	20,587	20,692	21,069	20,773	20,665	20,592	20,510
District of Columbia	2,742	2,765	2,751	2,972	2,728	2,807	2,939	3,135	3,003	2,780
Delaware	4,495	4,799	5,058	5,141	5,239	5,277	5,281	5,466	5,459	5,681
Florida	101,494	104,701	105,486	107,733	110,183	109,603	108,931	110,653	109,554	108,216
Georgia	36,724	37,382	39,182	42,013	42,977	43,660	43,118	44,921	46,154	45,984
Hawaii	6,013	6,103	6,194	6,406	6,640	6,773	6,730	6,788	6,852	7,000
Idaho	6,170	6,573	6,766	7,171	7,148	7,366	7,431	7,551	7,538	7,358
Illinois	61,518	61,898	63,773	64,956	66,301	66,557	65,198	66,369	65,130	64,959
Indiana	30,156	30,659	31,712	32,023	32,417	32,423	32,677	32,864	32,543	32,372
Iowa	16,254	16,457	16,744	16,852	16,944	17,594	17,526	17,531	17,175	16,911
Kansas	13,644	13,695	14,003	14,553	14,727	14,608	14,608	14,906	14,843	14,572
Kentucky	22,498	22,774	23,960	24,527	24,697	24,919	25,484	25,669	26,191	26,068
Louisiana	22,071	21,076	21,575	22,431	22,906	23,560	23,661	24,150	24,198	24,184
Massachusetts	35,459	35,919	37,105	37,201	37,736	37,478	35,711	37,198	36,481	36,097
Maryland	25,491	25,865	26,002	27,907	28,447	28,463	28,710	28,642	28,489	29,824
Maine	8,347	8,305	8,628	8,542	8,472	8,356	8,385	8,336	8,481	8,366
Michigan	52,128	52,819	53,501	55,831	53,580	54,009	55,167	55,617	53,248	52,067
Minnesota	24,502	24,622	25,505	26,801	26,990	26,901	27,028	28,019	27,835	27,770
Missouri	28,895	30,601	30,832	31,108	31,042	31,893	31,203	31,901	32,136	31,628
Mississippi	13,506	13,663	14,235	15,005	15,328	15,033	15,460	15,361	15,343	15,482
Montana	4,914	5,225	5,192	5,350	5,369	5,612	5,627	5,722	5,517	5,610
North Carolina	41,513	43,778	44,991	46,870	47,706	49,465	49,546	50,395	49,248	49,970
North Dakota	3,327	3,477	3,497	3,594	3,525	3,485	3,585	3,724	3,694	3,508
Nebraska	8,807	9,031	9,171	9,398	9,216	8,851	9,158	9,204	9,018	9,176
Nevada	—^§^	—	—	—	—	—	—	—	—	—
New Hampshire	7,002	7,233	7,280	7,448	7,714	7,618	7,742	7,971	7,745	7,886
New Jersey	46,552	46,971	48,590	49,194	49,083	49,495	48,838	49,687	49,268	49,960
New Mexico	8,247	8,272	8,486	8,775	8,984	9,021	9,006	8,851	8,815	8,728
New York	100,219	101,660	104,618	106,397	108,138	108,711	106,341	110,138	106,911	109,560
Ohio	58,765	60,115	60,763	61,490	63,211	62,975	61,175	62,348	60,801	62,802
Oklahoma	17,758	17,875	18,960	19,744	18,857	18,957	18,637	18,897	19,082	19,044
Oregon	18,499	18,682	19,112	19,376	20,370	19,909	19,727	20,636	20,035	20,458
Pennsylvania	73,045	73,803	75,923	77,186	77,243	77,601	77,931	79,562	76,689	77,562
Rhode Island	6,289	6,001	6,270	6,358	6,269	6,239	5,762	5,898	6,168	6,097
South Carolina	21,118	21,933	22,555	23,504	23,576	23,883	24,638	24,737	25,399	24,809
South Dakota	4,021	3,922	3,830	4,079	4,029	4,191	4,126	4,429	4,225	4,417
Tennessee	27,702	30,218	30,998	32,100	32,957	34,014	33,854	34,374	34,363	34,142
Texas	90,532	92,394	94,362	98,911	99,603	100,782	99,386	100,351	101,919	101,962
Utah	7,763	7,920	8,309	8,517	8,859	9,108	9,496	9,712	9,681	9,626
Virginia	33,484	34,571	35,351	37,244	37,308	37,846	36,402	37,277	36,744	38,151
Vermont	3,387	3,460	3,718	3,602	3,529	3,681	3,659	3,630	3,609	3,510
Washington	30,679	31,403	31,843	31,339	33,240	34,143	34,320	34,869	34,883	34,865
Wisconsin	27,929	27,869	28,645	29,475	29,967	30,174	30,575	31,413	30,572	30,638
West Virginia	10,741	11,071	11,250	11,546	11,296	11,352	11,231	11,638	11,336	11,327

*Invasive cancer excludes basal and squamous cell carcinomas of the skin except when these occur on the skin of the genital organs, and in situ cancers except urinary bladder.

^†^Data are compiled from cancer registries that meet the data quality criteria for all invasive cancer sites combined (all registries except Nevada, covering approximately 99% of the U.S. population). Registry-specific data quality information is available at https://www.cdc.gov/cancer/npcr/uscs/pdf/uscs-2013-technical-notes.pdf#nameddest=RegistriesPubCriteria.

^§^Counts are not presented for Nevada because data from Nevada did not meet USCS publication criteria for all the years from 2004 to 2013.

**TABLE 12:** Reported rate* of invasive^†^ cancer cases, all cancer sites combined, by state — United States, 2004–2013^§^

State	2004	2005	2006	2007	2008	2009	2010	2011	2012	2013
Alaska	511.2	481.2	486.2	471.6	479.5	460.5	458.7	430.7	404.0	410.4
Alabama	460.6	461.7	464.7	473.4	485.0	477.2	466.6	457.7	453.8	444.0
Arkansas	462.1	465.1	461.3	468.4	457.6	455.2	453.5	445.0	450.1	454.0
Arizona	418.1	408.7	413.1	419.1	405.9	415.7	394.8	399.8	384.7	370.6
California	451.6	448.1	446.1	455.5	451.0	445.6	437.2	425.6	414.0	402.8
Colorado	447.1	447.1	439.7	456.9	447.0	445.2	429.4	423.1	409.8	396.1
Connecticut	507.5	510.0	524.5	518.6	511.4	514.1	499.7	490.4	482.3	474.2
District of Columbia	487.4	487.0	488.7	523.8	477.2	482.2	494.6	516.6	482.4	445.2
Delaware	507.7	525.8	537.5	529.1	526.3	516.6	507.6	511.8	496.7	502.0
Florida	470.9	474.1	469.7	470.9	471.9	460.6	447.8	444.3	428.5	413.0
Georgia	476.6	468.9	473.0	490.3	485.5	480.1	459.5	468.3	465.0	450.3
Hawaii	435.2	430.2	427.4	434.2	438.7	438.9	425.6	421.8	418.1	419.8
Idaho	463.1	478.0	473.0	484.4	467.3	467.6	458.0	454.4	442.2	419.5
Illinois	492.8	490.0	500.1	501.6	503.5	497.8	479.9	480.8	463.8	454.9
Indiana	476.4	477.3	485.4	481.5	477.4	469.5	465.4	460.3	448.8	438.8
Iowa	485.3	487.6	490.4	487.7	485.1	497.0	489.5	484.4	468.0	456.1
Kansas	481.1	477.7	481.9	493.6	492.0	479.4	473.5	477.6	467.4	450.9
Kentucky	521.9	518.1	533.8	535.1	528.3	523.3	526.4	521.6	522.1	511.7
Louisiana	497.0	481.1	493.8	501.1	500.3	502.0	495.4	494.5	485.8	476.3
Massachusetts	514.3	517.0	529.8	523.3	522.0	509.5	477.9	489.3	471.9	457.5
Maryland	463.9	463.1	458.1	482.3	482.3	469.1	465.4	452.3	440.2	451.0
Maine	544.7	534.0	544.8	527.8	512.8	499.1	493.4	484.0	478.7	463.8
Michigan	504.2	504.5	504.2	517.1	489.8	485.8	489.1	485.1	456.8	440.1
Minnesota	481.2	475.7	483.3	496.5	488.5	476.9	471.3	477.7	462.8	451.8
Missouri	470.3	490.5	485.7	481.5	471.8	476.2	458.4	462.2	457.6	442.6
Mississippi	468.1	469.2	479.5	495.1	498.4	482.2	485.8	474.6	465.3	459.9
Montana	473.6	492.2	478.2	475.6	467.3	476.9	472.2	468.8	442.1	437.0
North Carolina	478.1	490.3	486.6	490.2	483.8	489.3	477.6	473.3	450.8	445.4
North Dakota	462.9	479.8	475.8	485.2	467.5	458.7	462.9	470.0	464.0	433.6
Nebraska	479.3	485.0	485.9	490.7	473.6	449.0	456.4	453.8	435.7	437.6
Nevada	—	—	—	—	—	—	—	—	—	—
New Hampshire	523.9	530.6	519.3	517.8	524.6	506.3	502.0	510.8	484.6	479.2
New Jersey	508.9	509.4	522.0	521.7	512.8	509.6	494.6	496.9	484.4	483.1
New Mexico	431.0	420.4	416.5	419.0	418.8	409.4	397.0	381.7	374.7	363.7
New York	496.2	499.5	511.0	513.1	514.8	510.0	492.1	501.2	479.8	484.3
Ohio	479.4	485.3	484.3	482.5	488.6	480.8	459.7	463.0	444.8	452.4
Oklahoma	477.6	475.0	494.7	505.9	474.8	467.8	452.4	451.5	449.5	440.3
Oregon	484.8	477.6	475.0	470.6	482.3	461.5	447.5	458.1	432.5	431.5
Pennsylvania	502.5	503.9	512.8	515.2	509.9	506.8	502.8	507.5	482.9	483.0
Rhode Island	530.8	508.5	526.6	532.4	519.1	512.6	468.7	476.6	488.9	479.4
South Carolina	484.2	490.4	485.8	489.0	475.0	469.2	472.1	463.4	459.8	436.9
South Dakota	476.5	459.9	440.3	460.9	448.2	459.6	443.2	467.5	439.6	450.1
Tennessee	449.7	479.2	478.8	483.3	484.0	489.9	476.6	472.9	463.6	450.9
Texas	467.9	464.3	457.7	465.3	454.4	445.8	427.3	417.6	411.5	399.4
Utah	426.4	419.7	423.4	419.2	421.7	419.9	424.5	422.9	408.4	393.2
Virginia	455.7	458.8	458.9	471.4	462.0	457.4	428.8	429.3	414.0	418.5
Vermont	499.5	501.2	529.0	501.3	479.1	492.8	481.0	469.9	457.0	437.1
Washington	505.3	504.3	496.9	475.4	491.6	490.1	479.6	476.3	463.3	450.3
Wisconsin	480.8	472.5	478.5	483.4	482.4	477.8	476.5	479.3	457.7	451.1
West Virginia	491.8	501.2	503.1	507.9	491.5	488.1	477.7	489.7	470.7	464.0
Wyoming	451.2	427.5	454.2	436.1	438.1	426.5	432.9	437.2	398.0	382.0

*Rates are the number of cases per 100,000 persons and are age-adjusted to the 2000 U.S. standard population (19 age groups – Census P25–1130). For more information, see USCS technical notes (https://www.cdc.gov/cancer/npcr/uscs/pdf/uscs-2013-technical-notes.pdf).

^†^Invasive cancer excludes basal and squamous cell carcinomas of the skin except when these occur on the skin of the genital organs, and in situ cancers except urinary bladder.

^§^Data are compiled from cancer registries that meet the data quality criteria for all invasive cancer sites combined (all registries except Nevada, covering approximately 99% of the U.S. population). Registry-specific data quality information is available at https://www.cdc.gov/cancer/npcr/uscs/pdf/uscs-2013-technical-notes.pdf#nameddest=RegistriesPubCriteria.

^¶^Rates are not presented for Nevada because data from Nevada did not meet USCS publication criteria for all the years from 2004 to 2013.

**TABLE 13:** Reported number of cancer deaths, all cancer sites combined, by state — United States, 2004–2013*

State	2004	2005	2006	2007		2008	2009	2010	2011	2012		2013
Alabama	9,756	9,913	9,899	10,025		10,182	10,289	10,196	10,233		10,274	10,328
Alaska	728	732	790	839	867		895	884	935		925	1,016
Arizona	9,618	9,820	9,918	10,133		10,081	10,271	10,678	10,690		11,085	11,347
Arkansas	6,304	6,361	6,177	6,388		6,526	6,513	6,475	6,497	6,540		6,688
California	53,700	54,729	54,140	55,011		54,686	55,990	56,453	56,448	57,675		57,714
Colorado	6,196	6,395	6,550	6,617		6,719	6,950	7,035	7,051		7,306	7,357
Connecticut	7,174	7,052	7,044	6,827		6,830	6,819	6,954	6,837		6,681	6,619
Delaware	1,827	1,799	1,780	1,852		1,912	1,813	1,909	1,905		1,935	1,905
District of Columbia	1,152	1,150	1,178	1,169		1,143	1,131	1,041	1,070		1,081	1,095
Florida	39,840	40,592	40,415	40,088		40,814	40,931	41,467	41,681		42,187	42,734
Georgia	14,313	14,358	14,474	14,983		14,621	15,139	15,435	15,602		16,020	16,417
Hawaii	2,088	2,169	2,171	2,214		2,194	2,244	2,266	2,278		2,284	2,332
Idaho	2,227	2,368	2,306	2,405		2,511	2,458	2,530	2,573		2,572	2,707
Illinois	24,289	24,250	24,083	24,113		24,298	24,182	24,070	24,006		24,562	24,491
Indiana	12,552	12,796	12,903	12,778		13,136	13,093	13,164	13,180		13,368	13,258
Iowa	6,340	6,453	6,359	6,376		6,424	6,249	6,358	6,481		6,438	6,509
Kansas	5,312	5,428	5,343	5,406		5,294	5,319	5,377	5,440		5,429	5,379
Kentucky	9,159	9,505	9,394	9,692		9,589	9,634	9,930	9,733		10,012	10,082
Louisiana	9,434	9,249	8,853	8,736		9,197	9,098	9,203	9,233		9,308	9,419
Maine	3,124	3,218	3,089	3,112		3,093	3,133	3,247	3,201		3,226	3,227
Maryland	10,168	10,371	10,350	10,179		10,360	10,412	10,268	10,249		10,524	10,608
Massachusetts	13,337	13,182	13,407	13,003		13,031	13,112	12,993	12,895		12,864	12,858
Michigan	19,653	20,094	20,192	20,087		20,211	20,257	20,620	20,420		20,496	20,367
Minnesota	9,093	8,823	9,079	9,176		9,446	9,580	9,612	9,489		9,424	9,601
Mississippi	5,983	6,065	6,236	6,001		6,165	6,130	6,271	6,278		6,496	6,527
Missouri	12,449	12,417	12,519	12,380		12,522	12,472	12,626	12,473		12,919	12,955
Montana	1,867	1,956	1,943	1,921		1,862	1,914	1,923	2,022		1,954	1,997
Nebraska	3,270	3,355	3,430	3,479		3,376	3,336	3,438	3,410		3,479	3,459
Nevada	4,119	4,238	4,225	4,331		4,404	4,461	4,529	4,605		4,610	4,817
New Hampshire	2,554	2,549	2,534	2,609		2,576	2,562	2,525	2,740		2,660	2,584
New Jersey	17,206	17,171	17,180	17,096		16,874	16,540	16,815	16,708		16,483	16,315
New Mexico	3,035	3,141	3,147	3,236		3,355	3,202	3,358	3,328		3,461	3,481
New York	36,100	35,555	35,283	35,485		35,351	35,215	35,431	35,469		35,881	35,735
North Carolina	16,477	16,724	17,318	17,478		17,453	17,513	18,060	18,284		18,405	18,589
North Dakota	1,265	1,302	1,387	1,264		1,354	1,243	1,269	1,321		1,253	1,286
Ohio	24,940	24,702	24,975	25,230		24,998	25,149	25,083	25,140		25,261	24,986
Oklahoma	7,269	7,446	7,491	7,727		7,657	7,639	7,831	7,997		8,040	8,039
Oregon	7,236	7,326	7,309	7,393		7,479	7,487	7,638	7,802		7,832	7,799
Pennsylvania	29,422	29,615	29,170	29,013		28,963	28,879	29,055	28,895		28,907	28,512
Rhode Island	2,418	2,291	2,250	2,213		2,227	2,220	2,266	2,170		2,148	2,326
South Carolina	8,348	8,652	8,853	8,867		9,199	9,123	9,356	9,543		9,728	9,745
South Dakota	1,555	1,612	1,570	1,612		1,570	1,503	1,655	1,665		1,630	1,577
Tennessee	12,586	12,995	13,051	13,161		13,162	13,482	13,593	13,562		13,765	13,953
Texas	33,937	34,291	34,938	35,074		35,712	35,591	36,717	37,351		38,142	38,412
Utah	2,445	2,520	2,615	2,572		2,492	2,555	2,810	2,746		2,876	2,971
Vermont	1,212	1,202	1,214	1,346		1,279	1,254	1,392	1,347		1,325	1,318
Virginia	13,384	13,877	13,828	14,009		13,983	14,122	14,078	14,374		14,294	14,414
Washington	10,989	11,048	11,055	11,568		11,618	11,922	11,874	12,002		11,951	11,928
West Virginia	4,694	4,617	4,613	4,690		4,605	4,786	4,685	4,782		4,684	4,718
Wisconsin	10,861	10,943	10,925	10,963		11,185	10,866	11,279	11,608		11,252	11,425
Wyoming	875	886	927	940		874	936	1,016	936		955	946

*Data are from the National Vital Statistics System (NVSS).

**TABLE 14:** Reported rate* of cancer deaths, all cancer sites combined, by state — United States, 2004–2013^†^

State	2004	2005	2006	2007	2008	2009	2010	2011	2012	2013
Alabama	204.6	204.7	199.8	198.5	197.3	196.3	190.7	187.4	184.8	182.1
Alaska	185.9	173.3	179.7	185.2	183.2	186.5	178.4	176.4	169.1	173.1
Arizona	167.4	164.2	160.0	158.8	153.2	152.0	152.6	148.3	148.1	146.4
Arkansas	208.3	207.5	196.6	201.0	201.1	198.0	193.7	191.2	188.6	189.6
California	169.4	169.2	164.8	163.9	158.9	158.8	155.8	151.8	150.7	146.6
Colorado	159.9	160.5	158.4	154.7	151.2	151.9	148.8	144.2	143.7	139.2
Connecticut	182.0	176.7	175.3	167.0	164.7	162.8	162.4	158.1	152.0	147.8
Delaware	208.0	199.1	190.6	193.3	192.7	178.0	184.4	179.8	176.2	167.1
District of Columbia	206.2	206.2	210.8	208.8	201.8	196.9	178.4	180.8	178.4	177.7
Florida	178.9	177.9	174.2	169.3	168.8	165.9	163.8	160.0	157.6	154.9
Georgia	196.3	190.8	184.9	186.1	175.7	176.3	173.9	171.0	169.6	168.1
Hawaii	150.2	151.4	148.2	147.5	142.0	141.3	140.5	138.6	134.4	134.9
Idaho	169.8	174.7	163.6	166.5	166.9	159.0	158.6	157.7	152.2	156.3
Illinois	194.9	192.4	188.9	186.6	185.0	181.7	177.9	174.7	175.4	171.7
Indiana	199.2	200.0	198.0	192.9	194.4	190.4	187.6	185.2	184.2	179.4
Iowa	181.8	183.1	178.6	176.5	176.4	169.6	170.7	172.4	167.9	168.2
Kansas	182.8	185.5	180.0	178.6	173.1	171.5	170.4	170.1	167.7	162.9
Kentucky	215.8	219.5	212.5	215.0	208.0	204.8	207.4	200.9	201.2	199.3
Louisiana	216.6	215.4	206.9	199.2	205.8	198.6	196.4	193.5	190.4	188.7
Maine	202.1	204.8	192.8	189.8	184.6	185.4	186.6	181.7	179.0	174.8
Maryland	190.9	190.7	187.4	180.9	180.6	176.7	170.2	166.0	165.9	163.0
Massachusetts	189.8	186.0	187.3	179.2	176.9	175.3	170.0	166.7	163.3	159.7
Michigan	191.1	192.7	190.8	186.4	184.5	182.0	182.0	177.4	174.3	170.2
Minnesota	176.8	169.1	171.0	168.4	170.3	168.7	166.1	160.5	155.6	155.1
Mississippi	210.2	211.3	213.2	201.9	202.8	199.7	200.4	196.7	200.0	196.5
Missouri	201.0	197.9	195.9	190.9	189.5	185.4	184.7	179.4	182.0	179.1
Montana	179.2	184.3	177.9	170.9	161.2	162.8	159.9	164.6	154.2	154.0
Nebraska	173.6	175.1	176.7	176.3	169.9	164.7	166.6	164.3	164.7	160.7
Nevada	192.5	191.7	185.5	181.3	179.8	175.6	173.5	170.3	163.7	164.9
New Hampshire	195.5	189.9	184.3	185.1	177.6	173.3	167.7	177.9	167.9	158.6
New Jersey	186.9	185.0	183.2	179.8	175.0	168.8	168.7	165.7	160.1	156.0
New Mexico	162.5	163.1	158.8	158.3	160.7	149.0	151.3	146.7	147.8	145.1
New York	177.7	173.5	171.1	169.7	166.6	163.9	162.1	159.6	159.4	155.5
North Carolina	194.0	191.6	191.8	187.3	181.3	177.0	177.9	174.8	170.4	167.7
North Dakota	166.2	169.9	179.8	162.3	171.2	155.6	156.1	160.4	150.6	150.8
Ohio	202.1	198.1	197.5	196.3	191.6	190.1	186.6	184.5	182.1	177.4
Oklahoma	196.0	198.0	195.3	198.5	193.0	189.3	190.4	191.2	189.4	185.4
Oregon	188.8	186.8	181.4	178.7	176.8	172.5	172.8	172.4	168.2	163.2
Pennsylvania	195.7	195.2	190.3	186.4	184.1	181.7	180.5	177.4	174.8	170.0
Rhode Island	196.4	186.5	181.8	177.8	177.5	173.7	176.7	168.5	163.3	173.9
South Carolina	195.9	198.3	195.0	188.4	190.3	182.8	182.6	182.3	179.1	174.0
South Dakota	177.3	181.7	172.7	174.1	167.9	158.4	169.8	168.6	162.1	154.1
Tennessee	208.4	210.6	205.0	201.6	197.1	197.7	194.6	189.1	187.8	185.4
Texas	182.6	179.5	176.7	172.3	170.4	164.8	165.1	162.5	160.3	156.9
Utah	141.0	141.0	140.2	133.6	125.8	124.9	133.0	125.6	128.8	127.9
Vermont	180.1	176.1	173.2	188.7	176.2	169.2	182.6	175.9	164.7	164.1
Virginia	189.0	190.6	185.8	184.1	179.4	176.4	171.7	170.8	165.2	162.3
Washington	184.9	181.4	175.9	179.4	175.9	175.1	169.7	166.9	161.8	156.3
West Virginia	212.3	207.6	204.0	204.3	197.9	203.3	196.6	199.7	191.1	190.5
Wisconsin	184.6	182.9	180.1	177.3	177.8	169.5	173.8	174.8	166.0	164.6
Wyoming	172.1	169.3	174.5	173.4	157.0	163.8	170.1	155.9	154.2	147.7

*Rates are the number of deaths per 100,000 persons and are age-adjusted to the 2000 U.S. standard population (19 age groups – Census P25–1130). For more information, see USCS technical notes (https://www.cdc.gov/cancer/npcr/uscs/pdf/uscs-2013-technical-notes.pdf).

^†^Data are from the National Vital Statistics System (NVSS).

National cancer surveillance data help public health officials track progress toward achieving the national cancer objectives set forth in *Healthy People 2020* (*18*). Differing rates of cancer by race, ethnicity, and state of residence indicate that for some populations, *Healthy People 2020* objectives have already been achieved, whereas objectives for other populations have not been met. For the national cancer burden to be reduced and *Healthy People 2020* targets to be met, behavioral and environmental factors that increase cancer risk must be reduced, and high-quality screening services, timely follow-up, and evidence-based treatments must be available and accessible to all persons. Cancer surveillance data can identify populations with high cancer rates that might benefit most from targeted cancer prevention and control efforts. Several effective evidence-based primary and secondary prevention measures, such as vaccination against infectious agents that cause cancer (i.e., hepatitis B virus and human papillomavirus), help with smoking cessation, and recommended cancer screening, when effectively implemented and sustained, could reduce the number of new cancer cases and prevent many cancer-related deaths (*19*). Evidence-based interventions can be implemented at both the individual level and the population level to reduce cancer risk factors, promote healthy living, and encourage cancer screening (*3*). The impact of these efforts can be monitored using cancer surveillance data.
